# Treatment context is associated with thrombocytopenia report phenotypes in lung cancer: an active-comparator analysis of FAERS and JADER

**DOI:** 10.3389/fimmu.2026.1970912

**Published:** 2026-10-02

**Authors:** Fan Yang, Qinliang Jiang, Zhineng Wei, Tongchen Hu

**Affiliations:** Department of Thoracic Surgery, Leshan People’s Hospital, Leshan, Sichuan, China

**Keywords:** FAERS, immune checkpoint inhibitor, JADER, lung cancer, pharmacovigilance, report classification, report phenotype, thrombocytopenia

## Abstract

**Background:**

Thrombocytopenia during immune checkpoint inhibitor treatment in lung cancer may be coded as immune, marrow suppression, coagulation, or non-specific. We tested whether coding-defined report phenotypes differed across co-reported treatment contexts.

**Methods:**

We analyzed FAERS (2015Q1–2026Q2) and JADER through Japanese fiscal 2025Q4 (31 March 2026). Strict primary lung cancer reports were grouped as ICI without chemotherapy (C1), ICI plus chemotherapy (C2), or chemotherapy without ICI (C3). Firth models compared mutually exclusive immune-like and marrow-like anchors. Complementary analyses evaluated the broad cohort, treatment date compatibility, C3 small-cell influence, phenotype variants, onset windows, treatment-variable ablation in elastic-net report phenotype models, and a 120-report random stratified blinded review with independent ratings, third-person adjudication, inverse-selection weighting, and bootstrap uncertainty.

**Results:**

FAERS included 1,362 immune-like or marrow-like anchors. Relative to C1, adjusted odds ratios (ORs) for immune-like versus marrow-like phenotype were 0.111 (95% CI, 0.069–0.173) in C2 and 0.014 (0.006–0.029) in C3; C3 was exploratory because only eight reports were immune-like. In 2,835 broad cohort reports, adjusted immune-like composition was 13.0% (95% bootstrap interval, 11.0%–15.1%) in C1 and 4.7% (3.4%–6.2%) in C2. Treatment date and stricter phenotype analyses were directionally consistent. JADER reproduced the C2 versus C1 direction but showed calibration shift; treatment-variable ablation reduced cross-database discrimination. Reviewers agreed on 111/120 reports (92.5%; 95% CI, 87.5%–96.7%; Cohen’s *κ*, 0.897; Gwet’s AC1, 0.901). In the locked 927-report frame, design-weighted final categories were 23.4% immune probable, 37.3% immune possible, 13.6% indeterminate, and 25.8% non-immune probable. Among 100 determinate reports, weighted AUROC was 0.892 (0.827–0.948), PR-AUC 0.956 (0.922–0.982), and calibration-in-the-large intercept 2.054 (1.707–2.432), indicating underprediction.

**Conclusion:**

Coding-defined thrombocytopenia report composition differed across treatment contexts. Blinded review supported reproducible structured category assignment and score-based report ordering, while calibration shift and treatment-variable ablation defined important limits. Findings support pharmacovigilance case prioritization and hypothesis generation, not clinical diagnosis, incidence estimation, patient-level etiologic attribution, or causal inference.

## Introduction

Lung cancer was the most frequently diagnosed cancer and the leading cause of cancer death worldwide in 2022 (1). Immune checkpoint inhibitors (ICIs) are now central to advanced non-small-cell lung cancer (NSCLC) and extensive-stage small-cell lung cancer (SCLC) treatment. These agents target programmed cell death protein 1 (PD-1), programmed death ligand 1 (PD-L1), or cytotoxic T-lymphocyte-associated protein 4 (CTLA-4). Treatment selection depends on histology, molecular profile, PD-L1 expression, performance status, and disease extent. Options include ICI monotherapy, dual-checkpoint blockade, or an ICI combined with platinum-based chemotherapy (2–4). This range of regimens expands treatment options but complicates attribution when cytopenias develop.

Immune activation can produce toxicities that resemble organ-specific autoimmunity. Major oncology guidelines emphasize early recognition because selected neurologic, cardiac, and hematologic events may require intervention even at lower grades (5–7). Hematologic immune-related adverse events are less common than dermatologic, endocrine, gastrointestinal, or pulmonary toxicities. They include immune thrombocytopenia (ITP), autoimmune hemolytic anemia, neutropenia, aplastic anemia, pure red-cell aplasia, thrombotic microangiopathy, and hyperinflammatory syndromes (8, 9). Evidence has largely come from case reports and small series with heterogeneous diagnostic assessment and treatment (10). More recent reviews have clarified their timing, diagnostic pitfalls, and management (11).

ICI-associated ITP is clinically important because severe platelet depletion may cause bleeding, prompt immunosuppression, and affect treatment interruption or rechallenge. A recent multicenter cohort described its frequency, risk factors, onset, recovery, and recurrence at scale (12). Pharmacovigilance and systematic review studies have also identified disproportionate ITP reporting with PD-1/PD-L1 inhibitors (13, 14). Broader FAERS studies have characterized thrombocytopenia and other hematologic or thrombotic events reported with ICIs (15, 16).

Thrombocytopenia during an ICI-containing regimen, however, is not necessarily immune-mediated. Trial-level evidence indicates that hematologic toxicity varies with the accompanying systemic regimen (17). Cytotoxic chemotherapy suppresses megakaryopoiesis, with consequences that depend on regimen, dose intensity, marrow reserve, infection, tumor involvement, and concomitant medications (18–21). Disseminated intravascular coagulation (DIC) represents a separate consumptive phenotype arising from sepsis, malignancy, tissue injury, or interacting causes (22). These etiologies require different investigations and treatment priorities and may lead to different decisions about continuing anticancer therapy.

Conventional spontaneous report studies usually compare a drug–event pair with a broad database background. This approach assesses disproportionate reporting but does not resolve the composition of thrombocytopenia coding patterns in comparable lung-cancer treatment contexts. We therefore constructed mutually exclusive, high-specificity coding-defined report anchors. We compared co-reported ICI and chemotherapy contexts while keeping DIC-like and undifferentiated reports outside the primary binary comparison. Broad cohort, timing, robustness, report-level onset, co-recorded seriousness, cross-database transport, model ablation, and clinical content analyses were used to define the scope and limits of the primary comparison.

## Materials and methods

### Study design and reporting framework

This retrospective observational study used deidentified individual case safety reports (ICSRs). The unit of analysis was a deduplicated report rather than a longitudinally followed patient. FAERS provided the primary active comparator analysis and model development data. JADER was analyzed independently for supportive report composition replication and cross-database model transport; estimates from the two databases were not pooled. Disproportionality analyses and reporting followed the READUS-PV statement and its explanation and elaboration (23, 24). Prediction model reporting followed TRIPOD (25). Original analysis objects were frozen before initial manuscript drafting; reviewer-requested analyses, sampling keys, code, and output hashes were separately frozen before interpretation of the revision results.

### Data sources and report processing

We processed quarterly FAERS ASCII files from 2004Q1 through 2026Q2. The primary window was 2015Q1–2026Q2, representing routine ICI use in lung cancer. The full 2004Q1–2026Q2 series was retained for sensitivity analyses. For JADER, we used the PMDA files released in July 2026 and imposed an inclusive cutoff at Japanese fiscal 2025Q4, which corresponds to 1 January–31 March 2026. Records assigned to fiscal 2026Q1 (1 April–30 June 2026) were excluded. Demographic, drug, indication, reaction, outcome, and therapy tables were linked at report level. FAERS drug indications were linked through the drug sequence identifier when available.

Reports on the official FAERS deletion list were removed. Within each CASEID, we retained the report with the latest FDA receipt date. Ties were resolved by selecting the numerically largest PRIMARYID. Reaction preferred terms (PTs) were deduplicated within each report. Drug names were normalized to prespecified active ingredients and grouped as ICI, cytotoxic chemotherapy, anti-vascular endothelial growth factor treatment, or other therapy. For JADER, the highest numeric report version within the cutoff was retained for each identifier. Japanese drug and reaction terms were harmonized with the frozen FAERS concepts.

### Lung cancer cohort and treatment contexts

Eligible indications had to identify primary lung cancer explicitly. Reports describing lung metastasis or secondary malignant neoplasm of the lung were excluded. Boundary-safe dictionaries distinguished NSCLC from SCLC; less specific terms were classified as other primary lung cancer. The main FAERS cohort required the target ICI or chemotherapy to have a primary suspect role. A sensitivity analysis also admitted secondary suspect roles.

We defined three mutually exclusive co-reported treatment contexts. C1 required a primary suspect ICI without co-reported cytotoxic chemotherapy. C2 required a primary suspect ICI and at least one co-reported cytotoxic chemotherapy agent. C3, the active comparator, required primary suspect cytotoxic chemotherapy without a co-reported ICI. These labels described report content and did not by themselves verify concurrent administration. Tyrosine kinase inhibitors and antibody–drug conjugates were not classified as cytotoxic chemotherapy. Anti-VEGF use was retained as a covariate. JADER used the same C1 and C2 definitions. Its C3 context was retained descriptively but was too sparse for the principal cross-database contrast.

### Treatment date compatibility

Treatment contexts were defined from co-reported suspect drugs and did not, by themselves, establish concurrent administration. We therefore profiled availability of the event date and ICI and chemotherapy start and end dates. Temporal compatibility analyses progressively required both treatment starts and an event date, both starts on or before the event, start dates within 30 or 90 days of one another, or documented overlap of the recorded ICI and chemotherapy intervals. No missing dates were imputed. C1 comparison reports were required to have an ICI start on or before the event date in the date-restricted analyses.

### Broad thrombocytopenia and coding-defined report anchors

The broad thrombocytopenia cohort included the MedDRA PTs thrombocytopenia, platelet count decreased, autoimmune thrombocytopenia, idiopathic thrombocytopenic purpura, immune thrombocytopenia, and immune thrombocytopenic purpura. Reports were then assigned to five mutually exclusive coding-defined categories (**Supplementary Table S1**). Immune-like reports contained broad thrombocytopenia plus at least one explicit immune thrombocytopenia PT and no marrow or DIC anchor. Marrow-like reports contained broad thrombocytopenia plus at least one prespecified marrow suppression PT and no immune or DIC anchor. DIC-like reports contained broad thrombocytopenia plus a DIC/coagulopathy PT and no immune or marrow anchor. Reports with at least two report pattern axes were mixed. Reports with broad thrombocytopenia but no immune, marrow, or DIC anchor were undifferentiated.

Only mutually exclusive immune-like and marrow-like anchors entered the primary composition cohort. DIC-like reports were retained as a competing report phenotype and were not collapsed into the binary outcome. Mixed and undifferentiated reports were excluded from the binary report composition estimate but retained in the broad cohort analysis. The immune-like anchor therefore represented explicit report coding, not a confirmed clinical diagnosis of ITP.

### Main report composition analysis

The primary estimand was the OR for an immune-like rather than marrow-like report phenotype across treatment contexts among anchor-defined reports. Firth penalized logistic regression limited sparse data and separation bias (26), with C1 as the reference. The FAERS model included report year, age and its missingness indicator, sex, reporter group, lung cancer subtype, ICI class/combination, and anti-VEGF use. A penalized likelihood ratio test first evaluated the three-level treatment-context term. We then estimated C2 versus C1, C3 versus C1, and C2 versus C3 contrasts with Holm adjustment. A mean bias-reduced logistic model served as an implementation orthogonal sensitivity analysis.

To express the association on an absolute report composition scale, adjusted probabilities were standardized by assigning every included report to each treatment context in turn and averaging the predictions. Ninety-five percent confidence intervals (CIs) were obtained from 5,000 coefficient simulations. Probability differences and ratios were interpreted as report composition estimands rather than population risks. JADER was modeled separately using the same anchor outcome and treatment-context definitions.

### Broad cohort multinomial analysis

To evaluate selection induced by conditioning on immune-like and marrow-like anchors, we retained the complete broad thrombocytopenia cohort. The outcome had four mutually exclusive states: immune-like, marrow-like, DIC/mixed, and undifferentiated. A penalized multinomial logistic model included treatment context and the available report-level covariates used in the primary analysis. We summarized the mean fitted class probability among reports observed in each treatment context. Ninety-five percent intervals used 1,000 bootstrap samples drawn within treatment context. This estimand described adjusted report composition; it was not a counterfactual treatment probability.

### C3 small-cell influence and stricter phenotype analyses

Because C3 contained only eight immune-like reports, we tabulated their exact immune-anchor PTs and case-level structured evidence. We repeated the adjusted model after leaving out each report, after single-case reclassification, and under three stress tests: retaining only a lexically strict immune PT subset, excluding a report with malignant progression evidence, and excluding reports with explicit non-thrombocytopenic immune event support. Additional strict phenotype analyses were restricted to healthcare professional reports, excluded literature reports, or excluded reports with prespecified infection/sepsis or malignant progression competing context terms. These analyses evaluated coding robustness and did not clinically validate the anchors.

### Reporting-dependency analyses

For each immune-like, marrow-like, and DIC-like outcome, we formed 2 × 2 tables within the strict lung cancer treatment report universe. Crude reporting odds ratios (RORs) and calendar-year-stratified Mantel–Haenszel RORs (MH-RORs) were calculated for every context comparison. A prespecified frequentist signal required at least five event reports and a lower 95% CI above 1. The Bayesian confidence propagation neural network generated information components (ICs) and IC025 values. Parameters were *α* = *β* = 2, *α*1 = *β*1 = *γ*11 = 1, with a dynamic *γ*. IC025 was defined as the posterior mean minus two posterior standard deviations. The implementation followed the information component framework (27) and was tested with an independent numeric fixture and zero-cell cases. IC025 greater than zero indicated reporting dependency, not causality.

### Report-level onset proxy and co-recorded seriousness

The onset proxy was calculated as the recorded event date minus the target treatment start date. Complete dates and non-negative intervals were required, and day 0 was retained. FAERS does not provide a separate event date for each PT. We therefore treated the measure as a report-level onset proxy rather than a patient-level time-to-event outcome. Empirical cumulative distribution functions, medians, and interquartile ranges (IQRs) summarized the distributions. The median difference and Cliff’s delta intervals used 5,000 bootstrap samples. Distributional separation was assessed with the Mann–Whitney test and Cliff’s delta.

We modeled onset-proxy availability with Firth logistic regression including phenotype, treatment context, and their interaction. For the missingness sensitivity analysis, inverse probability weights were truncated at the 1st and 99th percentiles. We did not perform Kaplan–Meier or Cox analyses.

In addition to the non-negative onset-proxy analysis, we repeated comparisons after restricting intervals to 0–180 or 0–365 days and after excluding day 0. These were report-level sensitivity analyses; no interval was interpreted as the latency of clinically confirmed immune thrombocytopenia.

Death, life-threatening outcome, and hospitalization were analyzed as co-recorded report outcomes. We calculated exact proportions and used adjusted Firth ORs to compare immune-like with marrow-like reports. Models included treatment context, report year, age, sex, and reporter group. Missing age was replaced with the median age in the analysis cohort, and a separate age missingness indicator was included in each model. Holm adjustment was applied across the three outcomes. These analyses neither attributed an outcome to thrombocytopenia nor estimated patient-level clinical risk.

### Structured evidence modules and report phenotype models

Sixteen prespecified non-leakage evidence modules captured co-recorded clinical domains. These were systemic inflammation, pulmonary and non-pulmonary infection, bleeding, thrombosis, shock or multiorgan failure, hepatic injury, and renal injury. Other modules covered gastrointestinal or mucosal toxicity, non-infectious respiratory injury, cardiovascular injury, neurologic or neuromuscular toxicity, endocrine/metabolic toxicity, dermatologic or musculoskeletal toxicity, explicit immune-mediated events, and constitutional deterioration. Definitions excluded the PTs used for immune-like, marrow-like, and DIC-like anchors. Module prevalence and Haldane-corrected ORs were descriptive.

Elastic-net logistic regression distinguished immune-like from marrow-like anchors. The primary model included age, report year, sex, reporter group, treatment context, ICI class/combination, dual-ICI use, anti-VEGF use, and chemotherapy class indicators. The secondary model added the 16 modules and an indicator for the absence of any non-leakage module. Numeric variables were median-imputed, accompanied by missingness indicators, and standardized. Categorical variables were imputed with the most frequent level and one-hot encoded. All preprocessing was fitted within each training fold. We used neither class weighting nor synthetic oversampling.

Model development used 604 FAERS anchor reports through 2024Q4, including 133 immune-like reports. Nested stratified cross-validation comprised five outer and four inner folds. The inner objective was negative log loss across seven regularization strengths and five elastic-net mixing parameters. The locked models used C = 0.3, l1 ratio = 0.25, the saga solver, and a maximum of 20,000 iterations. Temporal evaluation used 148 FAERS reports from 2025Q1 to 2026Q2, including 20 immune-like reports. Cross-database transport evaluation used all 376 JADER C1+C2 coding-defined anchors through 31 March 2026, including 145 immune-like reports. No refitting, feature selection, threshold reselection, or recalibration was performed. This evaluation assessed transport of report phenotype discrimination and calibration, not validation against clinical diagnoses.

Performance measures included AUROC, precision–recall AUC (PR-AUC), Brier score, log loss, calibration intercept, calibration slope, and equal frequency calibration bins. Bootstrap 95% CIs were calculated for aggregate performance. The final model was applied to 927 undifferentiated FAERS C1+C2 reports. Scores below 0.30, from 0.30 to below 0.70, and at least 0.70 were described as low, intermediate, and high immune-like scores. The thresholds were prespecified as an uncertainty band, were not derived from a clinical utility function, and had no diagnostic meaning. Sensitivity analyses used threshold pairs of 0.40/0.60 and 0.20/0.80 and restricted the cohort to reports with at least one non-zero module. Model-derived labels did not enter the treatment-context composition models.

### Model ablation

We evaluated two locked ablations using the same development set, nested folds, tuning grid, temporal set, and JADER set as the full models. The context-free model removed treatment context, ICI class/combination, dual-ICI use, anti-VEGF use, and all chemotherapy class indicators while retaining report covariates and the 16 evidence modules. The modules-only model retained only the 16 modules and the module zero indicator. Performance metrics and calibration were calculated identically to the full models. These ablations tested whether the report phenotype model retained discrimination without treatment and drug class information.

### Independent clinical review

Two reviewers independently assessed a ranked, model-selected proxy set of 39 reports and all 22 C1+C2 DIC-like reports. Review was limited to the public structured drug, reaction, indication, outcome, therapy, and reporter fields. The 39-report set was a deliberately enriched clinical content check and was not used to estimate diagnostic performance or prevalence. DIC-like report categories described the available structured content and did not establish mechanism. A third adjudicator resolved disagreements, and agreement statistics were calculated before adjudication.

Separately, we drew 120 undifferentiated reports using a fixed seed, with 20 reports from each cell defined by C1/C2 and within-context score tertiles. Two reviewers received separately ordered workbooks that omitted treatment-context labels, model probabilities, score ranks, tertile labels, and prior review results. Using only public structured fields, each reviewer independently assigned immune probable, immune possible, non-immune probable, or indeterminate. Before adjudication, we estimated exact agreement, Cohen’s *κ*, a linear-weighted *κ* sensitivity measure, and Gwet’s AC1; 95% CIs were obtained from 5,000 bootstrap samples drawn within the six locked sampling strata. A third reviewer adjudicated all disagreements after both independent files were locked. Final category proportions were weighted by the inverse probability of selection to the locked 927-report frame, with 2,000 within-stratum bootstrap samples. Exploratory score agreement used immune probable plus immune possible as review-positive and non-immune probable as review-negative in a primary determinate analysis; strict-probable and indeterminate-as-negative definitions were sensitivity analyses. We estimated weighted AUROC, PR-AUC, Brier score, calibration-in-the-large, and logistic recalibration intercept and slope. Review outcomes were not used to retrain or recalibrate the report phenotype model.

### Sensitivity analyses and statistical conventions

Sensitivity analyses expanded suspect roles, required lung-linked treatment records, restricted the data to healthcare professional reports, or excluded literature reports. Additional analyses were restricted to NSCLC or SCLC, excluded anti-VEGF use, or repeated comparisons across time and treatment strata. The full DRUG/PT fingerprint combined event date, age, sex, country, the complete sorted drug list with roles, and the complete sorted reaction PT list. Within each duplicate cluster, the most recent report was retained. Quantitative bias analysis examined non-differential and differential phenotype misclassification. An adversarial tipping analysis counted the minimum relabelings needed to reach the null. Exploratory ICI agent, chemotherapy class, and country strata were treated as descriptive when cells contained fewer than five reports.

All tests were two-sided. The primary family used a 0.05 significance level. Holm adjustment was applied to pairwise composition contrasts and seriousness outcomes, with false discovery rate control for exploratory strata where applicable. Analyses used R 4.5.3 with data.**Table 1**.18.4, duckdb 1.5.2, and logistf 1.26.1. Python 3.12.13 analyses used NumPy 2.5.2, pandas 3.0.5, and scikit-learn 1.8.0.

**Table 1:** Characteristics of anchor-defined thrombocytopenia reports in the primary FAERS analysis window.

Characteristic	C1 immune-like (*N* = 113)	C1 marrow-like (*N* = 144)	C2 immune-like (*N* = 40)	C2 marrow-like (*N* = 455)	C3 immune-like (*N* = 8)	C3 marrow-like (*N* = 602)
Report count and data availability						
Reports, *n*	113	144	40	455	8	602
Age available, *n* (%)	99 (87.6%)	110 (76.4%)	37 (92.5%)	363 (79.8%)	7 (87.5%)	514 (85.4%)
Age, median (IQR), years	72.0 (65.0–78.0)	68.0 (59.3–73.0)	72.0 (65.0–78.0)	69.0 (62.0–74.0)	68.0 (67.5–81.0)	68.0 (61.0–73.0)
Sex						
Female, *n* (%)	30 (26.5%)	41 (28.5%)	7 (17.5%)	109 (24.0%)	0 (0.0%)	152 (25.2%)
Male, *n* (%)	75 (66.4%)	67 (46.5%)	32 (80.0%)	259 (56.9%)	7 (87.5%)	380 (63.1%)
Unknown/not reported, *n* (%)	8 (7.1%)	36 (25.0%)	1 (2.5%)	87 (19.1%)	1 (12.5%)	70 (11.6%)
Reporter and lung cancer subtype						
Healthcare professional reporter, *n* (%)	87 (77.0%)	115 (79.9%)	34 (85.0%)	391 (85.9%)	5 (62.5%)	333 (55.3%)
NSCLC, *n* (%)	98 (86.7%)	84 (58.3%)	32 (80.0%)	252 (55.4%)	6 (75.0%)	324 (53.8%)
SCLC, *n* (%)	7 (6.2%)	19 (13.2%)	5 (12.5%)	163 (35.8%)	0 (0.0%)	94 (15.6%)
Other primary lung cancer, *n* (%)	8 (7.1%)	41 (28.5%)	3 (7.5%)	40 (8.8%)	2 (25.0%)	184 (30.6%)

Values are *n* (%) unless otherwise indicated. Percentages use the column total; age summaries use reports with non-missing age. Counts represent spontaneous safety reports, not treated patients or incidence estimates.

C1, ICI reported without cytotoxic chemotherapy; C2, ICI and chemotherapy co-reported; C3, chemotherapy reported without ICI; FAERS, FDA Adverse Event Reporting System; ICI, immune checkpoint inhibitor; IQR, interquartile range; NSCLC, non-small-cell lung cancer; SCLC, small-cell lung cancer.

## Results

### Cohort construction yielded distinct coding-defined report phenotypes

The strict FAERS treatment-context universe contained 38,556 C1, 14,332 C2, and 18,716 C3 reports in the primary window (**Figure 1**; **Supplementary Table S2**). The anchor comparison comprised 1,362 mutually exclusive reports, including 161 immune-like and 1,201 marrow-like reports. The immune-like/marrow-like counts were 113/144 in C1, 40/455 in C2, and 8/602 in C3. The remaining report phenotypes comprised 25 DIC-like reports across C1–C3, 28 mixed reports, and 1,420 undifferentiated reports. Of the latter, 927 C1+C2 reports entered report phenotype scoring. Among 1,043,448 latest-version JADER reports within the prespecified cutoff, 61,903 had a strict primary lung cancer indication, and 41,424 met one of the treatment-context definitions. JADER contributed 376 C1+C2 anchors, of which 145 were immune-like and 231 were marrow-like, and was analyzed separately.

**Figure 1:**
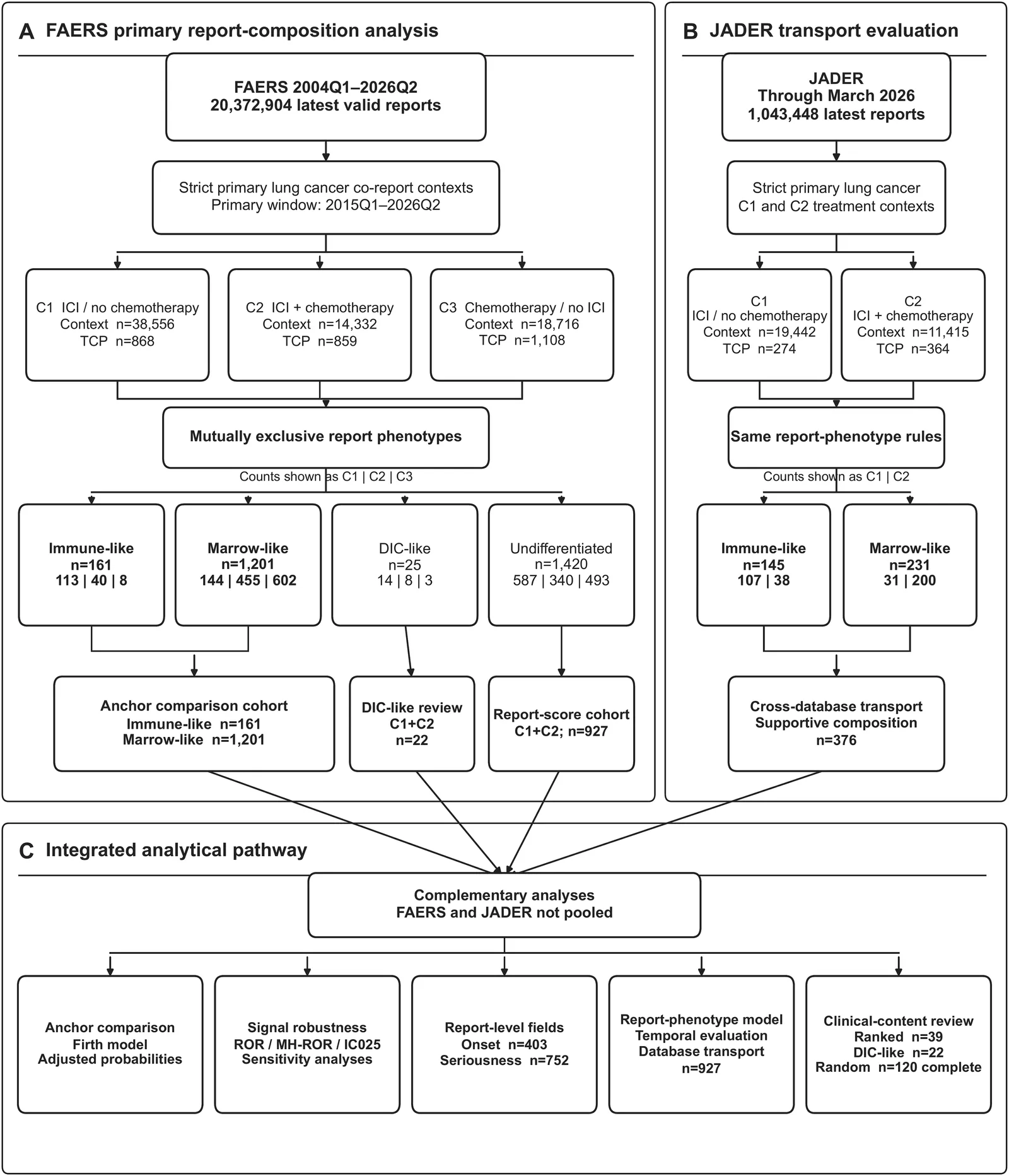
Study design, coding-defined report phenotypes, and integrated analytical workflow. **(A)** FAERS was used for the primary report composition analysis. After retaining the latest valid report version, co-reported treatment contexts were defined for 2015Q1–2026Q2 as ICI reported without cytotoxic chemotherapy (C1), ICI and chemotherapy co-reported (C2), and chemotherapy reported without ICI (C3). Reports with broad thrombocytopenia were assigned to mutually exclusive immune-like, marrow-like, disseminated intravascular coagulation (DIC)-like, mixed, or undifferentiated report phenotypes. The anchor comparison comprised 1,362 immune-like or marrow-like reports; 22 C1+C2 DIC-like reports underwent a descriptive clinical content review, and 927 C1+C2 undifferentiated reports entered report phenotype scoring. **(B)** The JADER release dated July 2026 was administratively censored at Japanese fiscal 2025Q4 (calendar 1 January–31 March 2026). Among 1,043,448 latest-version reports through that cutoff, 61,903 had a strict primary lung cancer indication; 376 C1+C2 immune-like or marrow-like reports formed the cross-database transport and supportive report composition set. **(C)** The integrated pathway comprised Firth regression, broad cohort multinomial analysis, disproportionality and robustness analyses, treatment date compatibility, report-level onset and co-recorded seriousness analyses, locked model transport and ablation, and clinical content review. FAERS and JADER were analyzed separately and were not pooled. Counts represent spontaneous safety reports rather than patients or incidence estimates.

Most anchor reports concerned older adults and men, although demographic completeness varied across contexts and phenotypes (**Table 1**). Median age ranged from 68 to 72 years across the six FAERS context-by-phenotype groups. NSCLC accounted for 86.7% of C1 immune-like reports and 58.3% of C1 marrow-like reports. The corresponding proportions were 80.0% for C2 immune-like reports and 55.4% for C2 marrow-like reports. Healthcare professionals submitted 77.0%–85.9% of C1/C2 anchor reports.

### Broad cohort analysis retained undifferentiated reports

The broad analysis retained 2,835 FAERS reports: 161 immune-like, 1,201 marrow-like, 53 DIC/mixed, and 1,420 undifferentiated. Adjusted mean fitted immune-like composition was 13.0% (95% bootstrap interval, 11.0%–15.1%) in C1, 4.7% (3.4%–6.2%) in C2, and 0.8% (0.3%–1.3%) in C3. The corresponding undifferentiated components were 67.6%, 39.6%, and 44.5%. Thus, the lower immune-like relative composition in C2 than in C1 was also present when non-anchor reports were retained, although most C1 reports remained undifferentiated.

### Reporting-dependency metrics separated immune-like, marrow-like, and DIC-like patterns

Reporting-dependency analyses produced a pattern distinct from the binary composition analysis (**Figure 2**). Relative to C1, marrow-like reporting was higher in C2 (MH-ROR, 7.095; 95% CI, 5.809–8.666; IC, 1.479; IC025, 1.299) and C3 (MH-ROR, 8.586; 95% CI, 7.136–10.332; IC, 1.299; IC025, 1.141). Immune-like reporting was similar in C2 and C1 (MH-ROR, 0.898; 95% CI, 0.615–1.311), but higher in C2 than C3 (MH-ROR, 4.924; 95% CI, 2.221–10.913; IC025, 0.271). No DIC comparison in the modern window met the concordant criteria for report count, confidence bound, and IC025. Across the modern and full windows, IC025 exceeded zero in 6 of 18 prespecified analyses and in none of the DIC-like comparisons (**Supplementary Figure S2** and **Supplementary Table S3**).

**Figure 2:**
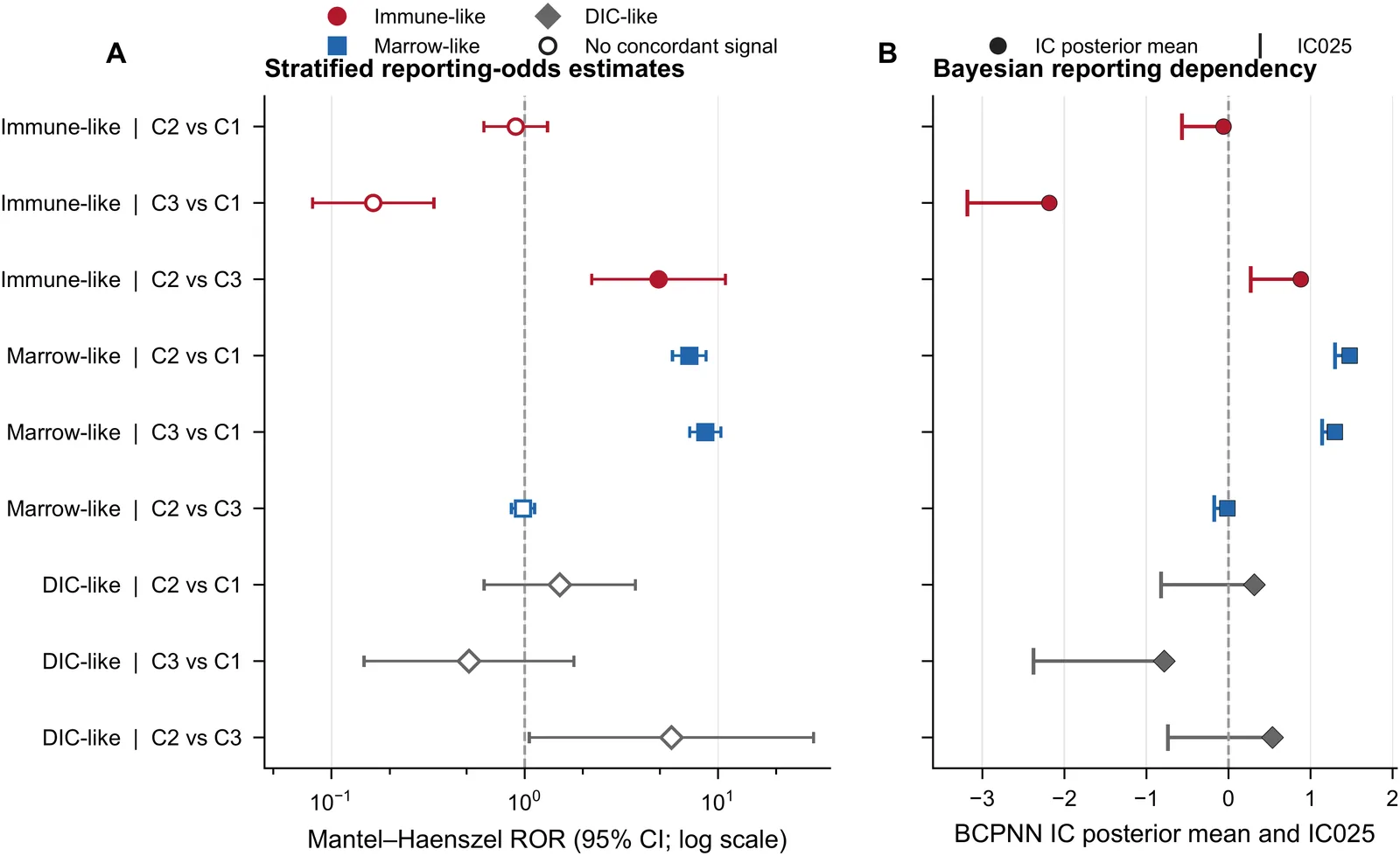
Reporting-dependency metrics distinguish immune-like, marrow-like, and DIC-like patterns in the primary analysis window. **(A)** Mantel–Haenszel-adjusted reporting odds ratios (MH-RORs) with 95% confidence intervals (CIs) for immune-like, marrow-like, and DIC-like outcomes across pairwise treatment-context comparisons in 2015Q1–2026Q2. Filled symbols denote analyses meeting the prespecified concordant signal criterion; open symbols denote the absence of a concordant signal. **(B)** Bayesian confidence propagation neural network estimates of the information component (IC). Symbols show posterior means and vertical ticks show IC025, the lower 95% credibility limit; the dashed vertical line denotes zero. These are reporting-dependency measures and do not estimate incidence or causal risk.

### Report phenotype composition differed across treatment contexts

The observed immune-like proportion fell from 44.0% in FAERS C1 to 8.1% in C2 and 1.3% in C3 (**Figure 3A**; **Table 2**). Phenotype composition differed across the three contexts (penalized likelihood ratio *χ*^2^ = 260.70, 2 *df*; *P* < 0.001). Relative to C1, the adjusted OR for an immune-like rather than marrow-like report was 0.111 (95% CI, 0.069–0.173; Holm-adjusted *P* < 0.001) for C2 and 0.014 (0.006–0.029; *P* < 0.001) for C3. The immune-like composition remained higher in C2 than in C3 (OR, 7.801; 95% CI, 3.570–19.154; *P* < 0.001) (**Figure 3B**).

**Figure 3:**
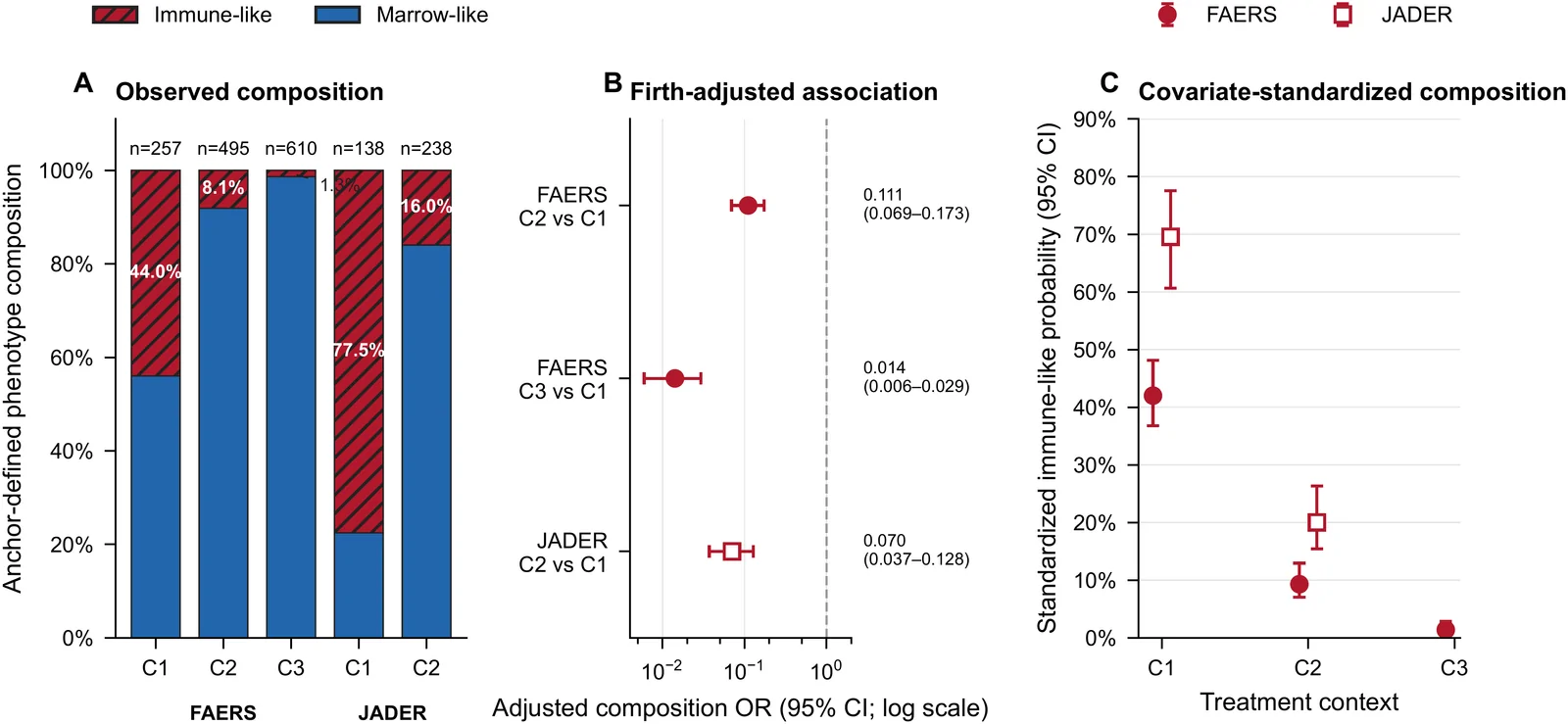
Treatment context is associated with the coding-defined composition of thrombocytopenia reports. **(A)** Observed proportions of anchor-defined immune-like and marrow-like phenotypes in FAERS C1–C3 and JADER C1–C2. Numbers above bars are report counts; percentages within or immediately beside the immune-like segments are immune-like proportions. **(B)** Firth logistic regression estimates for an immune-like rather than marrow-like phenotype, comparing C2 and C3 with C1 in FAERS and C2 with C1 in JADER. Points show odds ratios (ORs), and whiskers show 95% CIs. **(C)** Covariate-standardized probabilities of an immune-like phenotype by treatment context, shown separately for FAERS and JADER. Error bars show 95% CIs.

**Table 2:** Treatment context and the immune-like rather than marrow-like composition of thrombocytopenia reports.

Database	Context or contrast	Immune-like, *n*	Marrow-like, *n*	Observed immune-like, %	Standardized probability (95% CI)	Adjusted OR (95% CI)	*P*-value
Observed composition and covariate-standardized probabilities							
FAERS	C1	113	144	44.0%	42.0% (36.8–48.1)	–	–
FAERS	C2	40	455	8.1%	9.3% (7.0–13.0)	–	–
FAERS	C3	8	602	1.3%	1.4% (0.8–2.9)	–	–
JADER	C1	107	31	77.5%	69.6% (60.7–77.5)	–	–
JADER	C2	38	200	16.0%	20.0% (15.4–26.4)	–	–
Firth-adjusted contrasts: immune-like rather than marrow-like phenotype							
FAERS	C2 vs. C1	–	–	–	–	0.111 (0.069–0.173)	<0.001
FAERS	C3 vs. C1	–	–	–	–	0.014 (0.006–0.029)	<0.001
FAERS	C2 vs. C3	–	–	–	–	7.801 (3.570–19.154)	<0.001
JADER	C2 vs. C1	–	–	–	–	0.070 (0.037–0.128)	<0.001

Adjusted ORs estimate the odds of an immune-like rather than marrow-like report phenotype among immune-like and marrow-like anchor reports. FAERS models included report year, age with missingness indicator, sex, reporter group, lung cancer subtype, ICI class/combination, and anti-VEGF use; JADER was analyzed separately. Standardized probabilities were estimated by setting treatment context for every report and averaging predicted probabilities; uncertainty was assessed using 5,000 coefficient simulations. The FAERS global likelihood ratio test was *χ*^2^ = 260.70 with 2 degrees of freedom (*P* < 0.001). FAERS pairwise P-values were Holm-adjusted; the JADER C2 versus C1 test was database-specific. No cross-database pooling was performed.

C1, ICI reported without cytotoxic chemotherapy; C2, ICI and chemotherapy co-reported; C3, chemotherapy reported without ICI; OR, odds ratio.

Covariate-standardized immune-like probabilities were 42.0% (95% CI, 36.8%–48.1%) for C1, 9.3% (7.0%–13.0%) for C2, and 1.4% (0.8%–2.9%) for C3 (**Figure 3C**; **Supplementary Table S4**). The standardized differences were −32.7 percentage points (95% CI, −39.1 to −26.3) for C2 minus C1 and −40.6 percentage points for C3 minus C1.

JADER reproduced the direction of association, although its baseline composition differed from FAERS. Observed immune-like proportions were 77.5% in C1 (107/138) and 16.0% in C2 (38/238). The corresponding standardized probabilities were 69.6% (95% CI, 60.7%–77.5%) and 20.0% (15.4%–26.4%). The adjusted C2 versus C1 OR was 0.070 (95% CI, 0.037–0.128; *P* < 0.001), with a standardized difference of −49.5 percentage points (95% CI, −59.0 to −37.7). The databases were not pooled.

### Small-cell and treatment-timing analyses qualified the association

Five of the eight C3 immune-like reports contained the PT “immune thrombocytopenia” and three contained “immune thrombocytopenic purpura.” Leaving out one report at a time yielded adjusted C3 versus C1 ORs from 0.0119 to 0.0133, with convergence in every model. The lexically strict, malignant progression-excluded, and other immune evidence-excluded analyses yielded ORs of 0.0106, 0.0120, and 0.0081, respectively. These estimates were numerically stable but remained dependent on a small coding-defined cell.

The C2 versus C1 association was directionally consistent when restricted to healthcare professional reports (adjusted OR, 0.111; 95% CI, 0.066–0.181), after exclusion of literature reports (0.104; 0.060–0.174), and after exclusion of prespecified infection/sepsis or malignant progression competing context terms (0.094; 0.055–0.157).

Among 495 C2 anchors, an event date was available in 351, an ICI start date in 331, a chemotherapy start date in 311, and both start dates in 306. Both starts were on or before the event in 271 reports. Both treatment intervals could be constructed in 180 reports, and 168 had documented interval overlap. The strict overlap comparison included 153 C2 reports and yielded an adjusted C2 versus C1 OR of 0.060 (95% CI, 0.025–0.133). Analyses requiring available starts, starts before the event, or start dates within 30 or 90 days of each other were also directionally consistent. These restrictions supported timing compatibility in subsets but did not verify dosing or adherence.

### The context association persisted across design and misclassification analyses

The C2 versus C1 composition association remained below the null in every prespecified sensitivity analysis (**Figure 4A**; **Supplementary Table S5**). The adjusted OR was 0.145 (95% CI, 0.099–0.210) after inclusion of primary and secondary suspect roles and 0.111 (0.070–0.174) after full DRUG/PT fingerprint deduplication. Directional consistency was also retained in analyses restricted to healthcare professional reports, excluding literature reports, restricted to NSCLC or SCLC, or excluding anti-VEGF use. Full-fingerprint deduplication identified 32 clusters and removed 45 of 1,362 anchor reports. The resulting cohort retained 1,317 reports, with little change in the estimate (**Supplementary Figure S1**).

**Figure 4:**
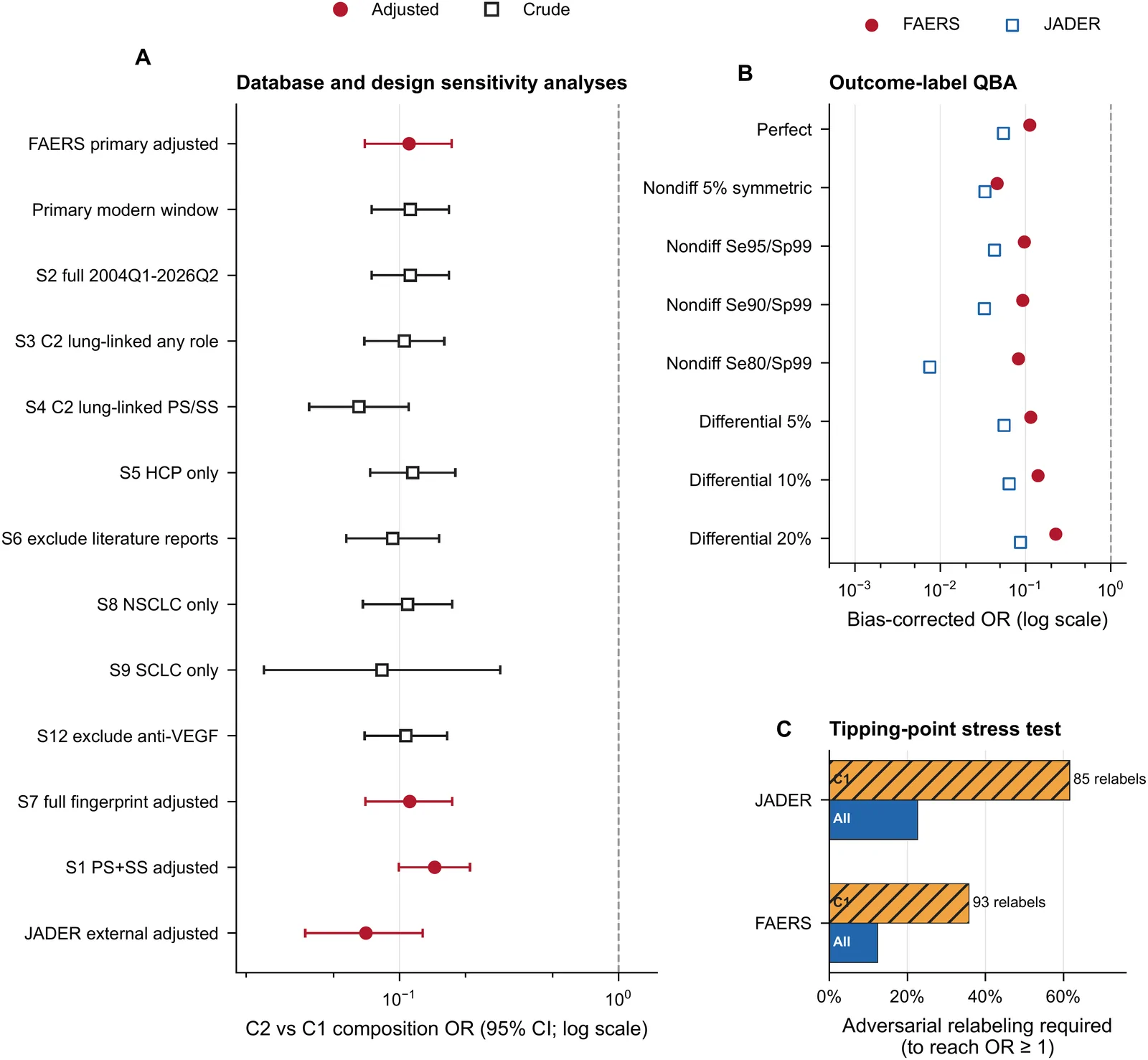
The treatment-context association is robust to design choices and outcome label error. **(A)** Primary and sensitivity estimates for the FAERS C2 versus C1 composition OR, together with the independently analyzed JADER estimate. Adjusted analyses are shown as filled circles and crude analyses as open squares; whiskers show 95% CIs. **(B)** Quantitative bias analysis under prespecified non-differential and differential phenotype misclassification scenarios. FAERS and JADER are shown separately; the dashed vertical line denotes the null OR of 1. **(C)** Adversarial tipping point analysis showing the proportion of all anchor reports and of C1 anchor reports that would have to be relabeled to move the C2 versus C1 association to the null. Labels report the corresponding number of relabelings.

Exploratory point estimates by ICI agent, chemotherapy class, and reporter country remained below 1 in all strata, although sparse strata yielded wide intervals and were treated descriptively (**Supplementary Figure S5**; **Supplementary Table S12**).

No prespecified feasible scenario in the quantitative bias analysis moved the association to the null (**Figure 4B**; **Supplementary Table S6**). The corrected FAERS OR was 0.226 under the adverse 20% differential misclassification scenario. Moving the crude C2 versus C1 association to the null required at least 93 relabelings, equivalent to 12.4% of all C1+C2 anchors or 35.8% of C1 anchors (**Figure 4C**). The association was therefore directionally robust to the tested errors, although residual classification error could not be excluded.

### Onset-proxy and co-recorded seriousness patterns differed between report phenotypes

A valid onset proxy was available for 84 of 153 immune-like (54.9%) and 319 of 599 marrow-like (53.3%) C1+C2 anchor reports (**Figure 5A**; **Table 3**). Median onset proxies were 27.5 days (IQR, 11.0–81.8) for immune-like reports and 10.0 days (IQR, 6.0–21.5) for marrow-like reports. The bootstrap median difference was 17.5 days (95% CI, 9.0–36.0). The full distributions differed in a two-sided Mann–Whitney test (*P* < 0.001), and Cliff’s *δ* was 0.390 (95% CI, 0.254–0.525). Inverse probability weighting gave medians of 42.0 and 10.0 days, retaining the same direction (**Figure 5B**). Onset availability varied by treatment context, but no phenotype-specific difference was statistically supported (**Supplementary Figure S3**; **Supplementary Table S7**).

**Figure 5:**
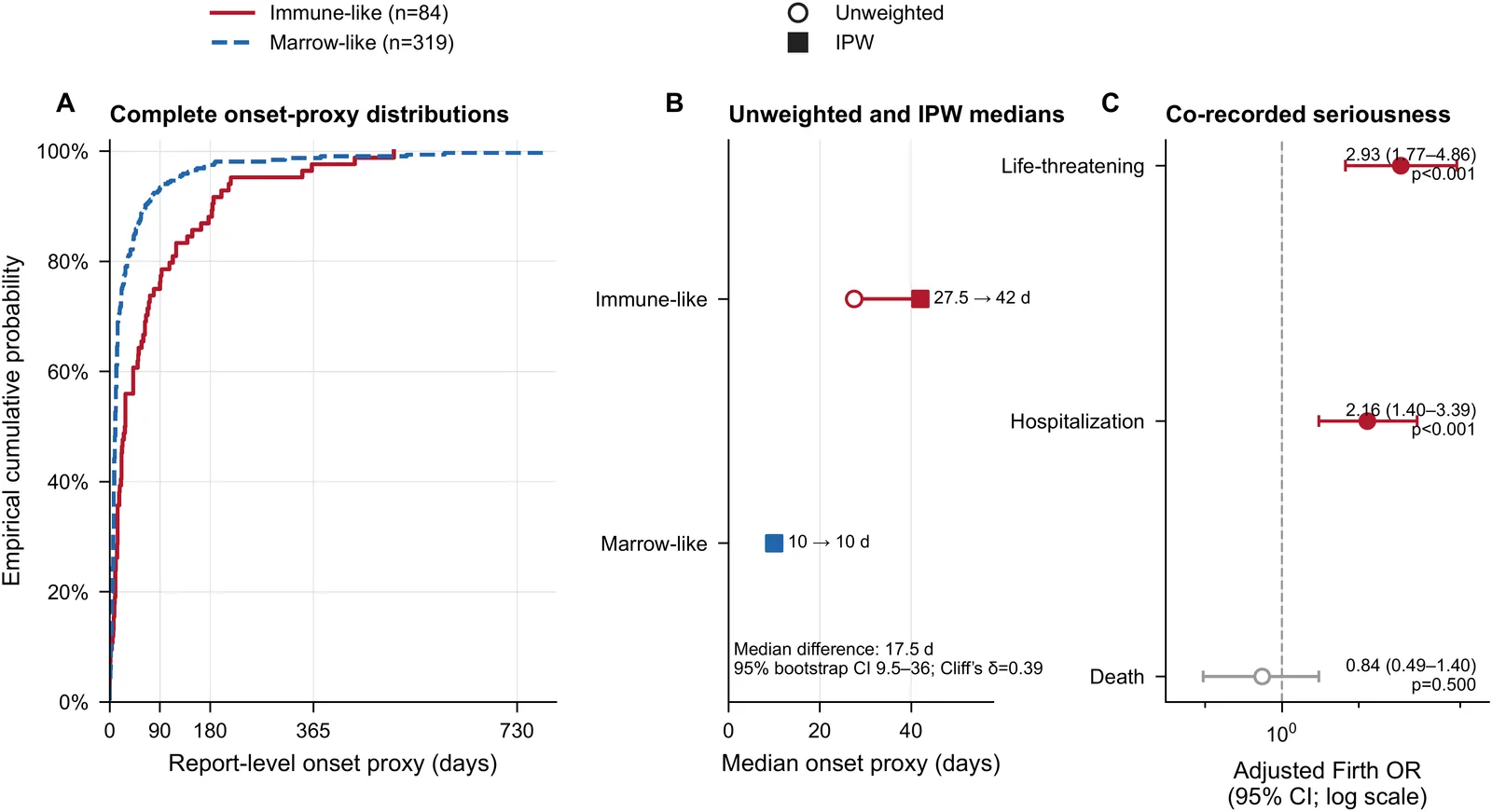
Immune-like and marrow-like reports differ in onset proxy and co-recorded seriousness. **(A)** Empirical cumulative distributions of all valid report-level onset proxies in FAERS anchor reports (immune-like, *n* = 84; marrow-like, *n* = 319; overall range, 0–780 days). **(B)** Unweighted and inverse probability-weighted (IPW) median onset proxies. The inset reports the unweighted median difference, bootstrap 95% CI, and Cliff’s delta. **(C)** Adjusted Firth ORs for co-recorded life-threatening outcome, hospitalization, and death in immune-like versus marrow-like reports. Models included treatment context, report year, age with median imputation and a missingness indicator, sex, and reporter group. Points and whiskers show ORs and 95% CIs; red indicates Holm-adjusted statistical significance. Onset values are report-derived proxies and are not patient-level time-to-event estimates.

**Table 3:** Report-level onset proxies and co-recorded seriousness by coding-defined report phenotype.

Domain/outcome	Immune-like	Marrow-like	Effect estimate (95% CI)	*P*-value	Sensitivity or scope
Report-level onset proxy in FAERS C1+C2 anchor reports					
Onset proxy available	84 (54.9%)	319 (53.3%)	–	–	Denominators include all C1+C2 anchor reports
Onset proxy, median (IQR), days	27.5 (11.0–81.8)	10.0 (6.0–21.5)	Median difference 17.5 days (9.0–36.0)	<0.001	IPW medians 42.0 vs. 10.0 days; IPW difference 32.0 days
Distributional effect	–	–	Cliff’s *δ* 0.390 (0.254–0.525)	<0.001	Positive values indicate later onset proxies for immune-like reports
Co-recorded seriousness in FAERS C1+C2 anchor reports					
Life-threatening	46 (30.1%)	87 (14.5%)	OR 2.927 (1.775–4.855)	<0.001	Immune-like vs. marrow-like co-recording
Hospitalization	98 (64.1%)	285 (47.6%)	OR 2.163 (1.396–3.390)	<0.001	Immune-like vs. marrow-like co-recording
Death	28 (18.3%)	106 (17.7%)	OR 0.836 (0.490–1.397)	0.500	Immune-like vs. marrow-like co-recording

Onset values are report-derived proxies, not patient-level time-to-event estimates. The *P*-value accompanying the median summary is from a two-sided Mann–Whitney test of the full distributions and is not a test of the median difference. The IPW sensitivity used 1st/99th percentile-truncated inverse probabilities of onset availability. Seriousness estimates are adjusted Firth ORs for co-recording in spontaneous reports and do not estimate clinical risk. Models included treatment context, report year, age with median imputation and a missingness indicator, sex, and reporter group. *P*-values were Holm-adjusted. Onset-proxy completeness, the availability model, and the IPW sensitivity are reported in **Supplementary Table S7** and **Supplementary Figure S3**.

Restriction to 0–180 days retained 385 reports; medians were 21 and 10 days, the median difference was 11 days (95% bootstrap CI, 5–19), and Cliff’s delta was 0.346 (0.198–0.485). Estimates remained directionally consistent in the 0–365-day analysis and after exclusion of day 0. These values describe report-level date fields, not clinical ITP latency.

Life-threatening outcome was co-recorded in 30.1% of immune-like reports and 14.5% of marrow-like reports (adjusted OR, 2.927; 95% CI, 1.775–4.855; Holm-adjusted *P* < 0.001). Hospitalization was co-recorded in 64.1% and 47.6%, respectively (OR, 2.163; 95% CI, 1.396–3.390; *P* < 0.001). Death was co-recorded in 18.3% and 17.7% (OR, 0.836; 95% CI, 0.490–1.397; *P* = 0.500) (**Figure 5C**). These outcomes were report attributes and were not attributed to the platelet phenotype.

### Structured evidence modules encoded reproducible phenotype differences

The 16 non-leakage modules identified report patterns that extended beyond the PTs defining each anchor (**Figure 6**; **Supplementary Table S8**). In FAERS, other explicit immune-mediated or autoimmune events (B15) were more common in immune-like reports than in marrow-like reports (15.0% versus 5.5%; descriptive OR, 3.045; 95% CI, 1.738–5.335). Immune-like reports less often included renal injury (B08), gastrointestinal or mucosal toxicity (B09), endocrine/metabolic toxicity (B13), or constitutional deterioration (B16). The respective ORs were 0.105, 0.156, 0.235, and 0.083. JADER reproduced the direction and confidence-bound separation for B08, B09, B13, B14, and B16, whereas B15 was not enriched among its immune-like anchors. These module associations were descriptive and were not interpreted as independent causal effects.

**Figure 6:**
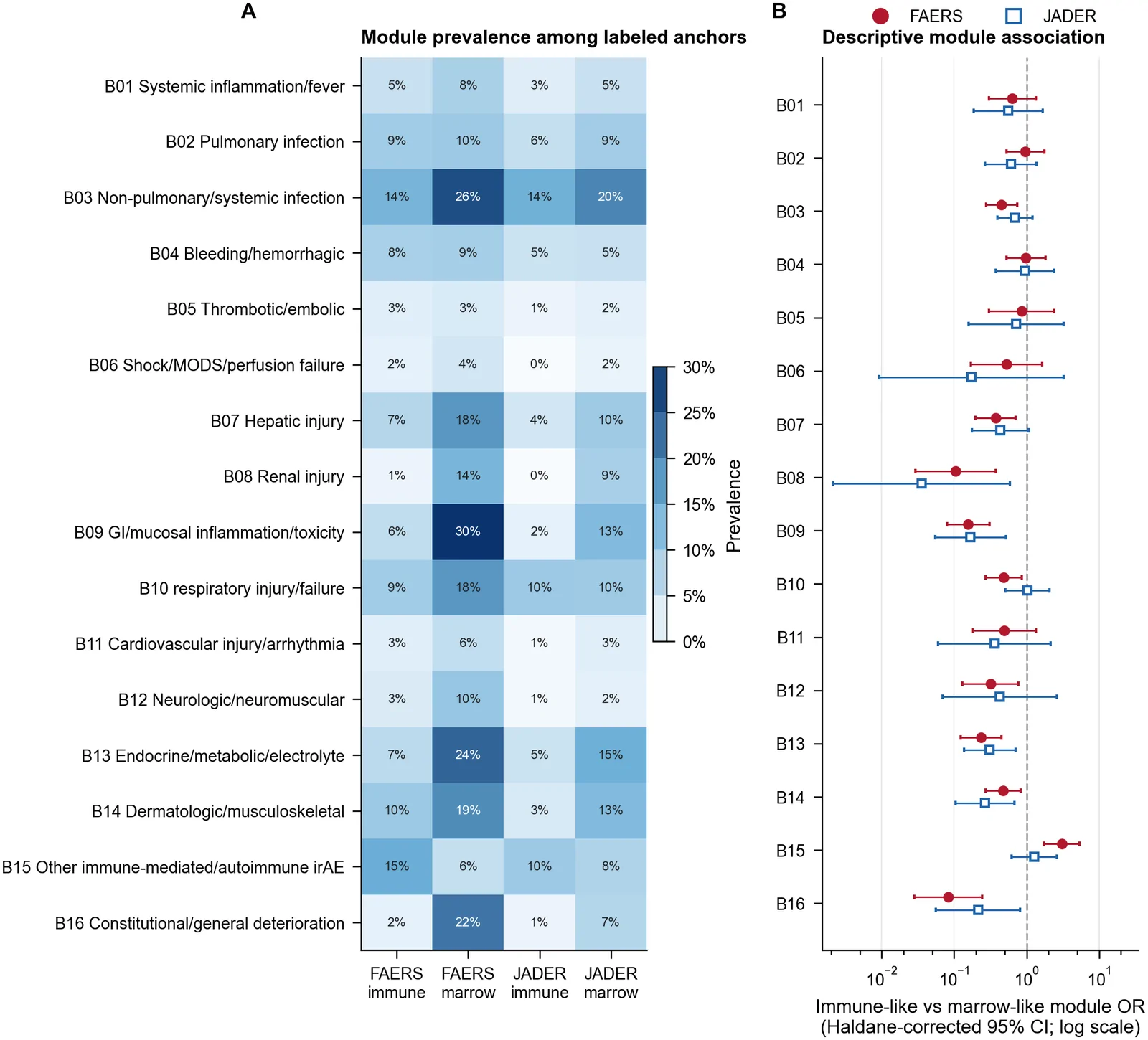
Sixteen structured evidence modules encode distinct immune-like and marrow-like phenotypes across databases. **(A)** Prevalence of each prespecified clinical evidence module among immune-like and marrow-like anchors in FAERS and JADER. **(B)** Descriptive immune-like-versus-marrow-like module ORs with Haldane-corrected 95% CIs, shown separately for FAERS and JADER. Module associations are descriptive and were not interpreted as independent causal effects.

### Report phenotype scores retained discrimination but not calibration across databases

Among 604 FAERS development reports, out-of-fold AUROC was 0.834 (95% CI, 0.796–0.871) for the primary model and 0.862 (0.827–0.892) for the secondary model. Corresponding PR-AUCs were 0.592 and 0.642 (**Figure 7A**; **Table 4**). In temporal evaluation, the secondary model had an AUROC of 0.830 (95% CI, 0.730–0.913), a PR-AUC of 0.454 (0.314–0.671), and a Brier score of 0.097 (0.077–0.119). The calibration intercept was −0.457 and the slope was 0.900; intervals were wide because only 20 immune-like reports were available.

**Figure 7:**
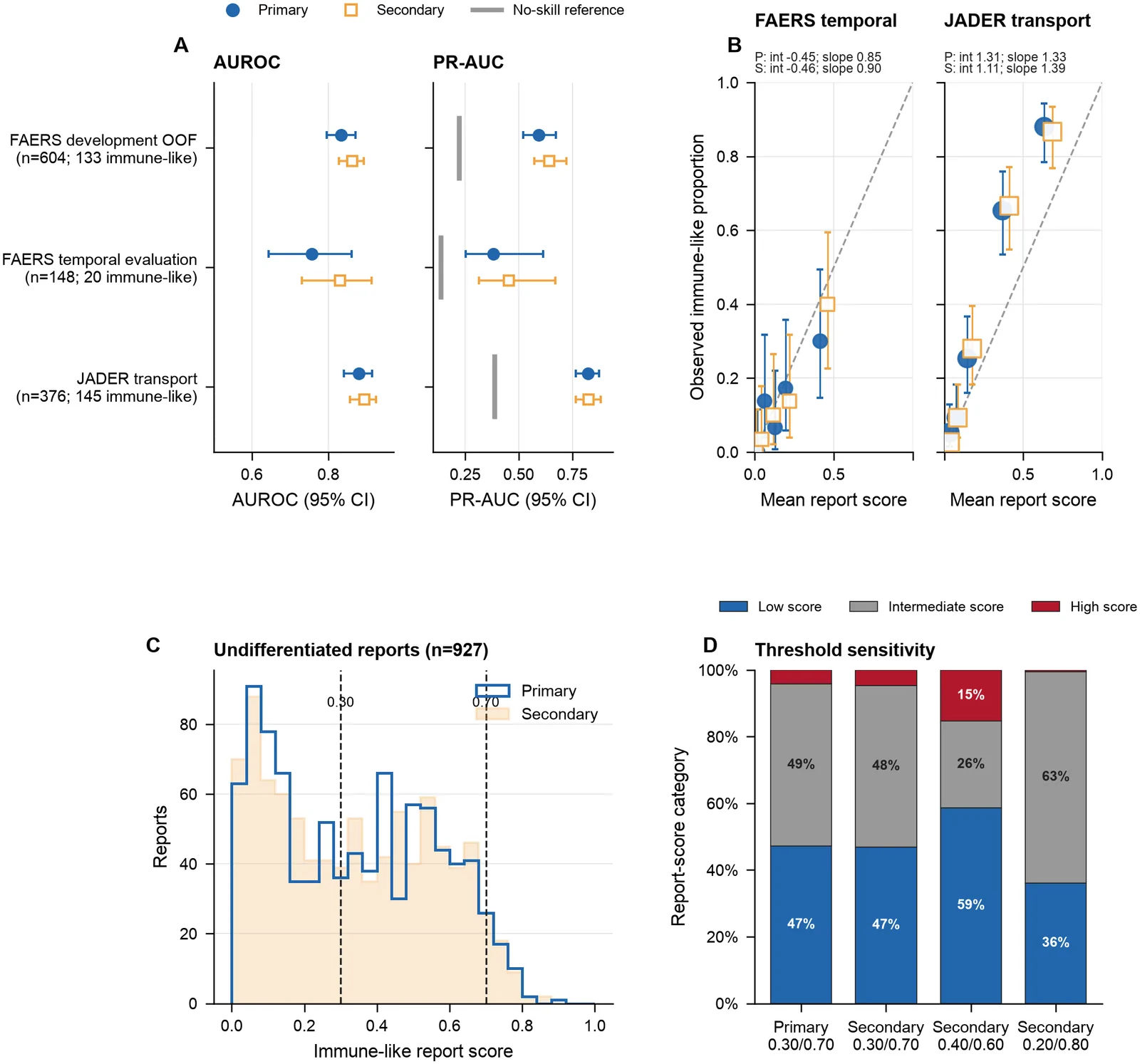
Report-phenotype model transport and scoring of the 927 undifferentiated reports. **(A)** Paired AUROC and precision -recall AUC (PR-AUC) estimates for the predefined primary and secondary report-phenotype models in FAERS out-of-fold development predictions, FAERS temporal evaluation, and JADER cross-database transport evaluation. Points and whiskers show estimates and bootstrap 95% CIs. **(B)** Calibration in the FAERS temporal and JADER transport sets using five equal-frequency bins per model. Points show observed immune-like report proportions and vertical whiskers show exact 95% CIs. **(C)** Distributions of primary- and secondary-model immune-like scores for the 927 undifferentiated FAERS C1+C2 reports. Dashed lines indicate the prespecified 0.30 and 0.70 uncertainty-band limits. **(D)** Classification of the 927 reports as low, intermediate, or high immune-like score under the primary and secondary models and threshold sensitivity analyses. These are report-classification metrics and score categories, not clinical diagnostic performance or diagnoses.

**Table 4:** Discrimination, calibration, and classification performance of the prespecified report phenotype models.

Model	*N*/immune-like *n* (%)	AUROC (95% CI)	PR-AUC (95% CI)	Brier score (95% CI)	Calibration intercept (95% CI)	Calibration slope (95% CI)
FAERS development OOF						
Primary	604/133 (22.0%)	0.834 (0.796–0.871)	0.592 (0.522–0.674)	0.125 (0.113–0.138)	0.025 (−0.159 to 0.251)	1.026 (0.857–1.238)
Secondary	604/133 (22.0%)	0.862 (0.827–0.892)	0.642 (0.572–0.723)	0.117 (0.104–0.130)	0.019 (−0.157 to 0.228)	1.007 (0.845–1.208)
FAERS temporal evaluation						
Primary	148/20 (13.5%)	0.756 (0.643–0.861)	0.382 (0.253–0.615)	0.103 (0.084–0.123)	−0.454 (−1.008 to 0.373)	0.854 (0.469–1.512)
Secondary	148/20 (13.5%)	0.830 (0.730–0.913)	0.454 (0.314–0.671)	0.097 (0.077–0.119)	−0.457 (−0.899 to 0.320)	0.900 (0.551–1.580)
JADER cross-database transport						
Primary	376/145 (38.6%)	0.880 (0.841–0.914)	0.823 (0.766–0.876)	0.161 (0.144–0.179)	1.313 (0.982 to 1.747)	1.328 (1.091–1.614)
Secondary	376/145 (38.6%)	0.894 (0.857–0.925)	0.827 (0.767–0.882)	0.147 (0.131–0.165)	1.105 (0.799–1.520)	1.385 (1.146–1.688)

Development performance uses out-of-fold predictions from nested five-fold cross-validation. Temporal evaluation comprised FAERS reports from 2025Q1 to 2026Q2; JADER was a cross-database transport set evaluated against the same coding-defined anchors. The primary model used structured treatment and report-context features; the secondary model additionally used the 16 prespecified non-leakage evidence modules and module-zero indicator. Locked classification thresholds and the corresponding sensitivity, specificity, and log-loss are reported in **Supplementary Tables S9–S11**.

AUROC, area under the receiver-operating characteristic curve; CI, confidence interval; OOF, out of fold; PR-AUC, area under the precision–recall curve.

In 376 JADER anchors, the secondary model achieved an AUROC of 0.894 (95% CI, 0.857–0.925), a PR-AUC of 0.827 (0.767–0.882), and a Brier score of 0.147 (0.131–0.165) without refitting or recalibration. Discrimination was similar for the primary model (AUROC, 0.880; 95% CI, 0.841–0.914). Calibration shifted upward for the secondary model (intercept, 1.105; 95% CI, 0.799–1.520; slope, 1.385; 95% CI, 1.146–1.688), in keeping with the higher observed immune-like prevalence in JADER (**Figure 7B**; **Supplementary Figure S4**).

At 0.30/0.70, 43 (4.6%) of 927 undifferentiated FAERS C1+C2 reports had a high immune-like score, 449 (48.4%) an intermediate score, and 435 (46.9%) a low score (**Figure 7C, D**; **Supplementary Tables S9–S11**). The corresponding primary model counts were 39, 450, and 438. At 0.40/0.60, the secondary model counts were 142, 241, and 544; at 0.20/0.80, they were 4, 588, and 335. Among 484 reports with at least one non-zero evidence module, the secondary model assigned 17 high, 202 intermediate, and 265 low scores. These categories were threshold dependent and did not constitute diagnoses.

### Treatment-variable ablation defined the model’s scope

The full secondary model achieved AUROCs of 0.862 in FAERS development, 0.830 in temporal evaluation, and 0.894 in JADER. Removing treatment-context and drug-class predictors reduced the corresponding AUROCs to 0.737, 0.760, and 0.720. The modules-only model yielded 0.709, 0.778, and 0.649. Among 927 undifferentiated reports, the mean full-model score was 0.462 in C1 and 0.119 in C2; the difference narrowed to 0.277 versus 0.216 with the context-free model and was absent with modules alone (0.250 versus 0.256). The model therefore captured substantial treatment and reporting-context rather than treatment-independent clinical evidence.

### Random stratified blinded review showed high agreement

Both reviewers completed all 120 reports. Before adjudication, they agreed on 111 reports (92.5%; stratified bootstrap 95% CI, 87.5%–96.7%), with Cohen’s *−* of 0.897 (0.831–0.954), linear-weighted *−* of 0.940 (0.901–0.974), and Gwet’s AC1 of 0.901 (0.835–0.956) (**Figure 8A**; **Supplementary Tables S25–S28**). The nine disagreements were confined to adjacent categories and were resolved by third-person adjudication after both independent ratings had been locked.

**Figure 8:**
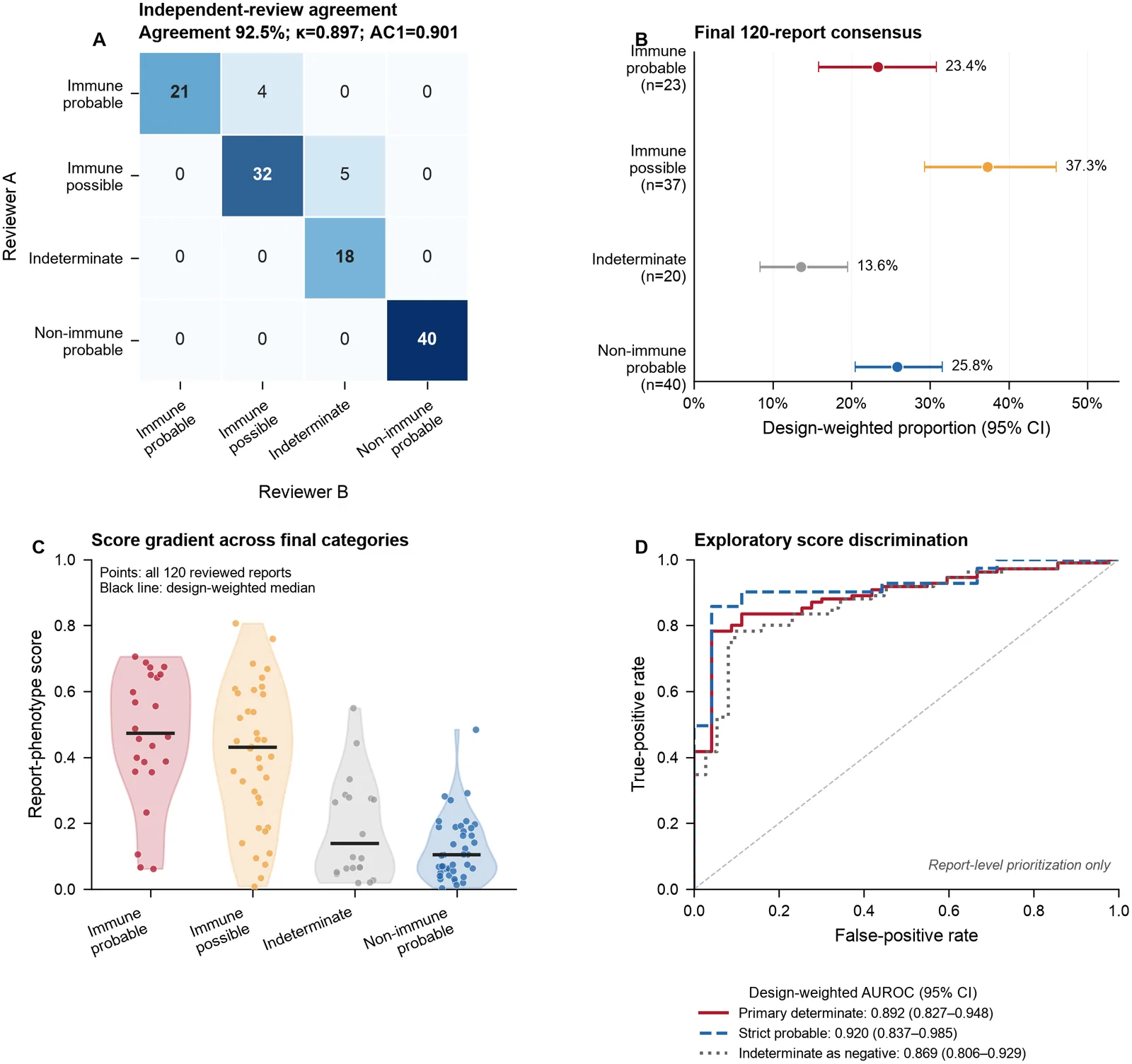
Independent clinical review of 120 randomly stratified undifferentiated reports. **(A)** Four-category cross-classification of the two independent ratings before adjudication; agreement statistics and stratified-bootstrap 95% CIs are shown. **(B)** Final consensus distribution after third-person adjudication, inverse-selection weighted to the locked 927-report frame; error bars denote within-stratum bootstrap 95% CIs. **(C)** Report-phenotype scores for all 120 reviewed reports by final consensus category; points show individual reports and horizontal markers show design-weighted medians. **(D)** Design-weighted receiver-operating-characteristic curves for the primary determinate definition, the strict-probable sensitivity definition, and the conservative indeterminate-as-negative definition. Review categories were based on available public structured report fields and are not confirmed diagnoses; performance estimates describe exploratory agreement and report prioritization, not clinical diagnostic validation.

Final consensus classified 23 reports as immune probable, 37 as immune possible, 20 as indeterminate, and 40 as non-immune probable. After inverse-selection weighting to the locked 927-report frame, the corresponding proportions were 23.4% (95% CI, 15.8%–30.8%), 37.3% (29.3%–46.1%), 13.6% (8.4%–19.5%), and 25.8% (20.5%–31.6%), respectively (**Figure 8B**; **Supplementary Tables S29, S30**). These are design-weighted structured report review categories, not prevalence estimates of clinically confirmed diagnoses. The earlier ranked 39-report and complete 22-report DIC reviews are retained in **Supplementary Figure S6 and Tables S13–S15** as enrichment and descriptive content checks.

### Exploratory score agreement supported report prioritization

The report phenotype score increased across the final review categories (**Figure 8C**; **Supplementary Table S31**). In the primary determinate analysis of 100 reports (60 review-positive and 40 review-negative), the design-weighted positive fraction was 70.2% (95% CI, 64.7%–75.6%), AUROC was 0.892 (0.827–0.948), PR-AUC was 0.956 (0.922–0.982), and Brier score was 0.256 (0.220–0.293). The calibration-in-the-large intercept was 2.054 (1.707–2.432); logistic recalibration yielded an intercept of 2.536 (1.938–3.666) and a slope of 1.345 (0.997–2.182), showing that the raw score underestimated the review-positive fraction in this enriched, score-stratified framework. AUROC was 0.920 (0.837–0.985) when only immune probable reports were review-positive and 0.869 (0.806–0.929) when indeterminate reports were treated as review-negative (**Figure 8D**; **Supplementary Table S32**). These exploratory estimates support score ordering for structured report prioritization, not calibrated clinical probabilities or diagnostic accuracy.

## Discussion

The relative frequency of coding-defined thrombocytopenia report phenotypes differed across co-reported treatment contexts in FAERS and JADER. This association was present in the selected immune-like versus marrow-like anchor comparison and in a four-state analysis that retained the broad thrombocytopenia cohort. Timing-restricted analyses reduced, but did not eliminate, uncertainty about concurrent exposure. The cross-database difference in baseline composition and calibration, together with the loss of model discrimination after treatment-variable ablation, shows that coding and reporting processes are integral to the observed pattern. The data therefore support a report-composition interpretation, not a biological treatment effect or patient-level etiologic attribution.

The onset-proxy distribution and co-recorded seriousness fields differed between coding-defined report phenotypes. These observations are not equivalent to the latency or severity of clinically confirmed ICI-associated ITP. Case reports and small series describe ITP after checkpoint inhibitors (28–32), but those clinically assessed patient timelines differ fundamentally from incomplete spontaneous-report date fields. The 0–180-day sensitivity analysis reduced the influence of very long intervals and retained the direction of the report-level comparison. The result should therefore be read only as separation between report records, not as an estimate of clinical ITP onset or a thrombocytopenia-specific effect on serious outcomes.

Differential diagnostic attribution is a plausible explanation for part of the association. A reporter evaluating thrombocytopenia after ICI exposure may preferentially select an immune-thrombocytopenia term, whereas a reporter evaluating a chemotherapy-containing report may record marrow-suppression terms. The strict-phenotype and competing-context analyses showed that the contrast was not explained by the tested lexical or contextual exclusions, but structured reports cannot recover platelet trajectories, marrow findings, infection severity, tumor infiltration, treatment response, or the reporter’s diagnostic reasoning (33, 34). The anchors should therefore be read as reproducible coding patterns, not verified clinical entities. The final random stratified review established high reproducibility of structured category assignment and supported score ordering, but the absence of full narratives, laboratory trajectories, and an independent patient-level gold standard precludes clinical diagnostic validation.

C2 reports cannot be treated as a simple extension of C1 reports. Chemotherapy-induced thrombocytopenia literature describes regimen-specific megakaryocytic suppression, cumulative marrow injury, infection, tumor involvement, and competing medications (35–38). These clinical mechanisms make a lower immune-like relative composition plausible, but the present data cannot distinguish underlying cause from treatment selection, diagnostic attribution, or coding behavior. The result does not indicate that chemotherapy protects against immune thrombocytopenia.

DIC-like reports formed a small competing set. DIC is a heterogeneous syndrome involving inflammation, coagulation activation, endothelial injury, and platelet consumption in settings that include sepsis and malignancy (39–41). The structured content of the 22 C1+C2 reports was categorized as treatment-related, septic, malignancy-related, multifactorial, or insufficient, but the complete set was too small and clinically sparse to estimate stable category proportions or establish mechanisms. Together with the absence of a concordant IC025 signal, this supports keeping DIC-like reports separate from both anchors and precludes individual drug ranking.

The active-comparator design addresses a different question from database-wide drug–event screening. Comparator choice changes the reference experience and can alter both the direction and magnitude of reporting bias (42). Restricting comparators to a shared therapeutic indication may improve clinical comparability (43). It can nevertheless introduce masking when reporting for the comparator is itself stimulated (44). Post-marketing studies also inherit source-specific missingness, selective reporting, confounding, and limitations in data fitness (45). Reporter type and report source can further affect which drug–event pairs cross a disproportionality threshold (46). We reduced these concerns through a strict primary lung cancer definition, separate treatment contexts, covariate adjustment, and a chemotherapy-only active comparator. IC and MH-ROR analyses, full-fingerprint deduplication, and phenotype-error analyses provided additional checks, but could not eliminate reporting bias.

The ablation analysis clarified the model’s scope. The full report phenotype model discriminated coding-defined anchors across time and database, but performance declined when treatment-context and drug-class variables were removed, and modules alone did not preserve cross-database discrimination. The model therefore captured how clinical evidence, treatment context, and reporting behavior jointly structure pharmacovigilance records. Its scores may support within-database case prioritization for manual assessment, but they are not treatment-independent etiologic probabilities. The JADER calibration shift further precludes transport of absolute FAERS probabilities or thresholds without database-specific recalibration and clinical validation (47–50).

Cross-database analysis revealed setting dependence. Recent FAERS–JADER studies have found database-specific signal magnitudes and only partial concordance after harmonization (51, 52). JADER had a substantially higher baseline immune-like composition than FAERS, although the C2 versus C1 direction was preserved. Differences in national drug utilization, reporter mix, regulatory reporting pathways, follow-up practices, PT selection, vocabulary harmonization, co-medication capture, and treatment selection could all contribute. These differences precluded pooling and explain why absolute FAERS scores cannot be transported to JADER without recalibration and clinical validation.

Several design features strengthened the analysis. The study began with a focused report-composition question rather than an unrestricted PT screen and used mutually exclusive, high-specificity anchors. Report phenotype scoring was kept separate from the primary treatment-context analysis to avoid circularity. Relative and absolute composition estimands were reported alongside ROR and IC metrics. Full DRUG/PT fingerprint deduplication, broad cohort modeling, treatment date restrictions, model ablation, quantitative misclassification analysis, and tipping analysis examined important sources of instability. These checks improved characterization of the reporting pattern but did not convert the design into causal or diagnostic research.

Several limitations constrain interpretation. Spontaneous reports lack a treated population denominator and are affected by underreporting, stimulated reporting, duplicate submission, indication error, incomplete co-medication records, and unmeasured confounding. Treatment selection may differ by molecular profile, PD-L1 expression, performance status, disease extent, tumor burden, prior lines, and regimen details that FAERS and JADER do not measure reliably. Co-reported drugs do not guarantee concurrent administration; date-compatible subsets reduce but do not remove this uncertainty. Anchor PTs are influenced by diagnostic and coding behavior, and the broad cohort remained largely undifferentiated. Laboratory values, platelet trajectories, marrow findings, bleeding grades, infection severity, and narrative reasoning were generally unavailable. The onset proxy was missing in approximately 46% of C1+C2 anchors, and the IPW analysis depends on measured predictors of availability. Seriousness fields indicate co-recording and cannot establish that thrombocytopenia caused hospitalization, life-threatening status, or death. None of the analyses estimates incidence, individual risk, confirmed etiology, or causality.

The model and review analyses have additional limits. C3 contained only eight immune-like reports, temporal evaluation included only 20 immune-like events, and JADER showed a material calibration shift. The earlier 39-report review set was ranked and enriched, and the 22-report DIC analysis is descriptive. The completed 120-report review used equal allocation across treatment-context and score-tertile strata; inverse-selection weighting recovers the locked 927-report frame under that design, but the review categories still depend on incomplete public structured fields and are not patient-level diagnoses. The large positive calibration-in-the-large intercept shows that the raw score underestimated the review-positive fraction in this score-stratified review framework and should not be interpreted as a calibrated probability. Even a full structured fingerprint may merge coincident independent reports or miss duplicates that differ in one component.

## Conclusion

In FAERS and JADER, coding-defined thrombocytopenia report composition differed across co-reported lung cancer treatment contexts. Broad cohort and timing-restricted analyses supported the direction of the association, whereas treatment-variable ablation and cross-database calibration differences showed that the report-generation process materially shaped the classification. These results may support pharmacovigilance case prioritization and hypothesis generation. They do not establish patient-level diagnosis, incidence, or a causal treatment effect.

## Data Availability

The original contributions presented in the study are included in the article/**Supplementary Material**. Further inquiries can be directed to the corresponding author.
