# Supplementary material for: Treatment context is associated with thrombocytopenia report phenotypes in lung cancer: an active-comparator analysis of FAERS and JADER
Source: Front Immunol. 2026 Oct 2;17:1970912. doi: 10.3389/fimmu.2026.1970912 (PMC13672329; doi:10.3389/fimmu.2026.1970912)
Supplement: Supplementary file 1 [file DataSheet1.docx]

**Supplementary Material**

**Contents**

| **Supplementary figures**  **Fig. S1** Full DRUG/PT deduplication **2**  **Fig. S2** Disproportionality and Bayesian IC **3**  **Fig. S3** Onset completeness/missingness **4**  **Fig. S4** Nested cross-validation and evaluation-set calibration **5**  **Fig. S5** Exploratory subgroup heterogeneity **6**  **Fig. S6** Legacy review concordance **7**  **Core analyses and legacy review**  **S1** Operational definitions **8**  **S2** Cohort and phenotype counts **10**  **S3** Disproportionality and Bayesian IC **11**  **S4** Standardized probabilities and contrasts **12**  **S5** Composition-association sensitivities **13**  **S6** Misclassification and tipping points **16**  **S7** Onset missingness and IPW **17**  **S8** FAERS/JADER evidence modules **19**  **S9** Model specification and nested CV **20**  **S10** Quintile calibration **22**  **S11** Score and threshold analyses **23**  **S12** Exploratory subgroup heterogeneity **24**  **S13** Legacy review agreement **25**  **S14** 39-report proxy case review **27**  **S15** 22-report DIC-like case review **31** | **Reviewer-requested analyses**  **S16** Broad four-state counts **34**  **S17** Standardized four-state composition **35**  **S18** C3 small-cell stress tests **36**  **S19** Strict phenotype and competing context **37**  **S20** Treatment and event date availability **38**  **S21** Treatment-date compatibility **39**  **S22** Model ablation performance **40**  **S23** Score distribution after ablation **43**  **S24** Onset-window sensitivities **44**  **Final blinded review and adjudication**  **S25** Two-reviewer agreement **45**  **S26** Pre-adjudication cross-classification **46**  **S27** Class-specific agreement **47**  **S28** Nine disagreements for adjudication **48**  **S29** Weighted final consensus **49**  **S30** Consensus by sampling stratum **50**  **S31** Score by final review category **51**  **S32** Score-review agreement **52**  **S33** Third-person adjudication **53** |
| --- | --- |

**Supplementary Figures**

**Figure S1. Full DRUG/PT fingerprint deduplication sensitivity analysis.** (A) Primary crude, full-fingerprint-deduplicated crude, and full-fingerprint-deduplicated Firth-adjusted C2-versus-C1 composition ORs with 95% CIs. (B) Immune-like and marrow-like anchor counts before and after full-fingerprint deduplication. (C) Distribution of duplicate-cluster sizes among the 32 identified clusters. DRUG/PT fingerprints incorporated the complete available drug and preferred-term lists for each report; 45 reports were removed in the sensitivity cohort.

**Figure S2. Expanded stratified disproportionality and validated Bayesian information-component analyses.** (A) MH-RORs with 95% CIs for immune-like, marrow-like, and DIC-like outcomes across both the modern and full analysis windows and all pairwise treatment-context comparisons. Filled symbols denote analyses meeting the prespecified signal criterion. (B) IC posterior means and IC025 values for the same analyses; the dashed vertical line denotes zero. This figure expands Figure 2 by adding the full database window.

**Figure S3. Completeness and missingness sensitivity analyses for the onset proxy.** (A) Proportions of reports with a valid onset proxy in C1 and C2, stratified by immune-like and marrow-like phenotype; points and whiskers show exact 95% CIs and labels show valid/total counts. (B) Prespecified Firth model of valid onset-proxy availability, including phenotype, treatment context, and their interaction. Points and whiskers show ORs and 95% CIs; red indicates Holm-adjusted statistical significance. (C) Unweighted and IPW median onset proxies by phenotype.

**Figure S4. Nested cross-validation and evaluation-set calibration details.** (A–C) AUROC, PR-AUC, and Brier score across the five outer folds for the primary and secondary models. (D) Calibration intercepts and slopes with 95% CIs in the FAERS temporal evaluation and JADER cross-database transport sets. P and S denote the primary and secondary report-phenotype models, respectively; dashed and dotted vertical lines denote the ideal intercept of 0 and slope of 1.

**Figure S5. Exploratory heterogeneity of the C2-versus-C1 report-phenotype composition association.** Composition ORs with 95% CIs across (A) ICI-agent strata, (B) chemotherapy-class strata, and (C) reporter-country strata. Red circles show strata meeting the prespecified inferential criteria and therefore display adjusted estimates; open gray squares show sparse strata presented descriptively with crude estimates. All subgroup analyses are exploratory.

**Figure S6. Detailed concordance of the two independent clinical reviews.** Reviewer A-by-reviewer B confusion matrices for (A) the 39-report proxy set and (B) the 22 DIC-like reports. Cells contain report counts and shading represents the within-row proportion. (C) Category-specific agreement, with labels showing the number of exact agreements over the reviewer-A category total.

**Supplementary Tables**

**TABLE S1
Operational definitions of treatment contexts, coding-defined report phenotypes, and clinical-review categories.**

| **Domain** | **Category** | **Operational definition** | **Analytical role** |
| --- | --- | --- | --- |
| **Treatment contexts** | | | |
| Treatment context | C1: ICI only | Strict primary lung-cancer report with a primary-suspect ICI and no cytotoxic chemotherapy co-reported | Primary reference context |
| Treatment context | C2: ICI plus chemotherapy | Strict primary lung-cancer report with a primary-suspect ICI and ≥1 cytotoxic chemotherapy drug co-reported | Primary combination context |
| Treatment context | C3: chemotherapy only | Strict primary lung-cancer report with primary-suspect cytotoxic chemotherapy and no ICI co-reported | Active comparator |
| **Mutually exclusive coding-defined report phenotypes** | | | |
| Phenotype | Immune-like | Broad thrombocytopenia plus ≥1 explicit immune thrombocytopenia PT; no marrow or DIC anchor | Positive anchor for model; explicit outcome for composition analyses |
| Phenotype | Marrow-like | Broad thrombocytopenia plus ≥1 marrow-suppression PT; no immune or DIC anchor | Negative anchor for model; explicit outcome for composition analyses |
| Phenotype | DIC-like | Broad thrombocytopenia plus a DIC/coagulopathy PT; no immune or marrow anchor | Competing phenotype; excluded from binary anchor model |
| Phenotype | Mixed | At least two report-level evidence axes present | Excluded from primary binary model; described separately |
| Phenotype | Undifferentiated | Broad thrombocytopenia with no immune, marrow, or DIC anchor | Report-score target; not a training class |
| **Independent clinical-review categories** | | | |
| Review category | Immune probable | Explicit immune or autoimmune features, or highly specific immune-mediated PTs, with compatible temporal or mechanistic support and no stronger competing explanation | 39-report proxy set; final consensus after independent review and adjudication when required |
| Review category | Immune possible | An immune mechanism remained plausible, but structured evidence was incomplete or a competing explanation could not be excluded | 39-report proxy set; final consensus after independent review and adjudication when required |
| Review category | Non-immune probable | A stronger non-immune explanation was present, such as cancer progression, infection or sepsis, marrow toxicity, or another drug effect | 39-report proxy set; final consensus after independent review and adjudication when required |
| Review category | Indeterminate | Available report fields did not support reliable report-phenotype classification, including isolated thrombocytopenia without sufficient temporal or mechanistic evidence | 39-report proxy set; final consensus after independent review and adjudication when required |
| Review category | Sepsis related | Infection or sepsis was judged the dominant explanation for the DIC/coagulopathy phenotype | DIC-like set; final consensus after independent review and adjudication when required |
| Review category | Malignancy related | Malignancy progression or tumor-related coagulopathy was judged the dominant explanation | DIC-like set; final consensus after independent review and adjudication when required |
| Review category | Treatment-related immune/coagulation | A treatment-related immune or coagulation toxicity was judged the most plausible explanation | DIC-like set; final consensus after independent review and adjudication when required |
| Review category | Multifactorial | At least two plausible mechanisms were present and no single cause was clearly dominant | DIC-like set; final consensus after independent review and adjudication when required |
| Review category | Insufficient information | Structured report information was insufficient to assign a report-phenotype category | DIC-like set; final consensus after independent review and adjudication when required |

*PT, MedDRA preferred term. Phenotypes are report-level operational constructs and are not equivalent to confirmed clinical diagnoses.*

**TABLE S2 Final treatment-context and coding-defined report-phenotype counts in FAERS and JADER.**

| **Database** | **Analysis window** | **Context** | **All context reports** | **Immune-like** | **Marrow-like** | **DIC-like** | **Mixed** | **Undifferentiated** |
| --- | --- | --- | --- | --- | --- | --- | --- | --- |
| **FAERS 2015Q1–2026Q2** | | | | | | | | |
| FAERS | 2015Q1–2026Q2 | C1 | 38556 | 113 | 144 | 14 | 10 | 587 |
| FAERS | 2015Q1–2026Q2 | C2 | 14332 | 40 | 455 | 8 | 16 | 340 |
| FAERS | 2015Q1–2026Q2 | C3 | 18716 | 8 | 602 | 3 | 2 | 493 |
| **FAERS 2004Q1–2026Q2** | | | | | | | | |
| FAERS | 2004Q1–2026Q2 | C1 | 38611 | 113 | 144 | 14 | 10 | 587 |
| FAERS | 2004Q1–2026Q2 | C2 | 14378 | 40 | 455 | 8 | 16 | 341 |
| FAERS | 2004Q1–2026Q2 | C3 | 25702 | 9 | 883 | 7 | 9 | 699 |
| **JADER through March 2026** | | | | | | | | |
| JADER | Through March 2026 | C1 | 19442 | 107 | 31 | 5 | 3 | 128 |
| JADER | Through March 2026 | C2 | 11415 | 38 | 200 | 4 | 13 | 109 |
| JADER | Through March 2026 | C3 | 10567 | 3 | 498 | 13 | 15 | 459 |

*Counts use the final V1.5.1 cohort definitions. FAERS and JADER were analyzed separately.*

**TABLE S3 Complete disproportionality and Bayesian information-component analyses.**

| **Phenotype** | **Comparison** | **a/b/c/d** | **Crude ROR (95% CI)** | **Year-stratified MH-ROR (95% CI)** | **P value** | **IC** | **IC025** | **Concordant signal** |
| --- | --- | --- | --- | --- | --- | --- | --- | --- |
| **FAERS 2015Q1–2026Q2** | | | | | | | | |
| Immune-like | C2 vs C1 | 40/14292/113/38443 | 0.952 (0.664–1.366) | 0.898 (0.615–1.311) | 0.581 | -0.060 | -0.567 | No |
| Immune-like | C3 vs C1 | 8/18708/113/38443 | 0.145 (0.071–0.298) | 0.164 (0.080–0.339) | <0.001 | -2.183 | -3.180 | No |
| Immune-like | C2 vs C3 | 40/14292/8/18708 | 6.545 (3.063–13.987) | 4.924 (2.221–10.913) | <0.001 | 0.882 | 0.271 | Yes |
| Marrow-like | C2 vs C1 | 455/13877/144/38412 | 8.746 (7.244–10.560) | 7.095 (5.809–8.666) | <0.001 | 1.479 | 1.299 | Yes |
| Marrow-like | C3 vs C1 | 602/18114/144/38412 | 8.865 (7.385–10.642) | 8.586 (7.136–10.332) | <0.001 | 1.299 | 1.141 | Yes |
| Marrow-like | C2 vs C3 | 455/13877/602/18114 | 0.987 (0.872–1.117) | 0.979 (0.853–1.123) | 0.764 | -0.012 | -0.173 | No |
| Dic-like | C2 vs C1 | 8/14324/14/38542 | 1.538 (0.645–3.666) | 1.517 (0.615–3.740) | 0.357 | 0.315 | -0.819 | No |
| Dic-like | C3 vs C1 | 3/18713/14/38542 | 0.441 (0.127–1.536) | 0.514 (0.147–1.797) | 0.289 | -0.783 | -2.378 | No |
| Dic-like | C2 vs C3 | 8/14324/3/18713 | 3.484 (0.924–13.134) | 5.741 (1.055–31.233) | 0.029 | 0.537 | -0.735 | No |
| **FAERS 2004Q1–2026Q2** | | | | | | | | |
| Immune-like | C2 vs C1 | 40/14338/113/38498 | 0.950 (0.662–1.364) | 0.898 (0.615–1.311) | 0.581 | -0.062 | -0.569 | No |
| Immune-like | C3 vs C1 | 9/25693/113/38498 | 0.119 (0.061–0.235) | 0.164 (0.080–0.339) | <0.001 | -2.326 | -3.275 | No |
| Immune-like | C2 vs C3 | 40/14338/9/25693 | 7.964 (3.863–16.418) | 4.924 (2.221–10.913) | <0.001 | 1.114 | 0.507 | Yes |
| Marrow-like | C2 vs C1 | 455/13923/144/38467 | 8.730 (7.231–10.540) | 7.095 (5.809–8.666) | <0.001 | 1.477 | 1.298 | Yes |
| Marrow-like | C3 vs C1 | 883/24819/144/38467 | 9.504 (7.963–11.343) | 8.623 (7.165–10.378) | <0.001 | 1.102 | 0.970 | Yes |
| Marrow-like | C2 vs C3 | 455/13923/883/24819 | 0.919 (0.819–1.030) | 0.972 (0.847–1.114) | 0.687 | -0.078 | -0.234 | No |
| Dic-like | C2 vs C1 | 8/14370/14/38597 | 1.535 (0.644–3.659) | 1.517 (0.615–3.740) | 0.357 | 0.314 | -0.821 | No |
| Dic-like | C3 vs C1 | 7/25695/14/38597 | 0.751 (0.303–1.861) | 0.524 (0.152–1.810) | 0.301 | -0.292 | -1.483 | No |
| Dic-like | C2 vs C3 | 8/14370/7/25695 | 2.044 (0.741–5.637) | 5.550 (1.052–29.283) | 0.031 | 0.417 | -0.785 | No |

*The concordant signal criterion required the prespecified frequentist and Bayesian conditions. IC025 is the lower 95% credibility limit of the information component. These are reporting-dependency measures, not incidence or causal-risk estimates.*

**TABLE S4
Covariate-standardized probabilities, probability differences, and probability ratios for the immune-like report phenotype.**

| **Estimand** | **Measure** | **Context/contrast** | **Estimate (95% CI)** | **Standardization N** | **Uncertainty method** |
| --- | --- | --- | --- | --- | --- |
| **FAERS** | | | | | |
| P(immune \| set context=C1) | Standardized probability | C1 | 42.0% (36.8–48.1) | 1362 | 5000 coefficient simulations |
| P(immune \| set context=C2) | Standardized probability | C2 | 9.3% (7.0–13.0) | 1362 | 5000 coefficient simulations |
| P(immune \| set context=C3) | Standardized probability | C3 | 1.4% (0.8–2.9) | 1362 | 5000 coefficient simulations |
| C2 minus C1 | Standardized probability difference | C2 vs C1 | −32.7 percentage points (95% CI, −39.1 to −26.3) | 1362 | 5000 coefficient simulations |
| C2 divided by C1 | Standardized probability ratio | C2 vs C1 | 0.222 (95% CI, 0.162 to 0.315) | 1362 | 5000 coefficient simulations |
| C3 minus C1 | Standardized probability difference | C3 vs C1 | −40.6 percentage points (95% CI, −46.7 to −35.0) | 1362 | 5000 coefficient simulations |
| C3 divided by C1 | Standardized probability ratio | C3 vs C1 | 0.033 (95% CI, 0.018 to 0.069) | 1362 | 5000 coefficient simulations |
| C2 minus C3 | Standardized probability difference | C2 vs C3 | 7.9 percentage points (95% CI, 5.1 to 11.6) | 1362 | 5000 coefficient simulations |
| C2 divided by C3 | Standardized probability ratio | C2 vs C3 | 6.673 (95% CI, 3.015 to 13.520) | 1362 | 5000 coefficient simulations |
| **JADER** | | | | | |
| P(immune \| set context=C1) | Standardized probability | C1 | 69.6% (60.7–77.5) | 376 | 5000 coefficient simulations |
| P(immune \| set context=C2) | Standardized probability | C2 | 20.0% (15.4–26.4) | 376 | 5000 coefficient simulations |
| C2 minus C1 | Standardized probability difference | C2 vs C1 | −49.5 percentage points (95% CI, −59.0 to −37.7) | 376 | 5000 coefficient simulations |
| C2 divided by C1 | Standardized probability ratio | C2 vs C1 | 0.288 (95% CI, 0.216 to 0.400) | 376 | 5000 coefficient simulations |

*All estimands are mathematical contrasts of fitted report-phenotype probabilities and must not be interpreted as incidence, an individual diagnosis, or clinical event risk.*

**TABLE S5 Complete composition-association sensitivity analyses.**

| **Sensitivity analysis** | **Comparison** | **Immune/marrow cells** | **N** | **OR (95% CI)** | **Minimum cell** | **Method** |
| --- | --- | --- | --- | --- | --- | --- |
| **Prespecified** | | | | | | |
| Primary modern window | C2 vs C1 | 40/455 vs 113/144 | 752 | 0.112 (0.075–0.168) | 40 | Crude |
| Primary modern window | C3 vs C1 | 8/602 vs 113/144 | 867 | 0.017 (0.008–0.035) | 8 | Crude |
| Primary modern window | C2 vs C3 | 40/455 vs 8/602 | 1105 | 6.615 (3.067–14.271) | 8 | Crude |
| S2 full 2004Q1-2026Q2 | C2 vs C1 | 40/455 vs 113/144 | 752 | 0.112 (0.075–0.168) | 40 | Crude |
| S2 full 2004Q1-2026Q2 | C3 vs C1 | 9/883 vs 113/144 | 1149 | 0.013 (0.006–0.026) | 9 | Crude |
| S2 full 2004Q1-2026Q2 | C2 vs C3 | 40/455 vs 9/883 | 1387 | 8.625 (4.149–17.932) | 9 | Crude |
| S3 C2 lung-linked any role | C2 vs C1 | 36/436 vs 113/144 | 729 | 0.105 (0.069–0.160) | 36 | Crude |
| S3 C2 lung-linked any role | C3 vs C1 | 8/602 vs 113/144 | 867 | 0.017 (0.008–0.035) | 8 | Crude |
| S3 C2 lung-linked any role | C2 vs C3 | 36/436 vs 8/602 | 1082 | 6.213 (2.860–13.499) | 8 | Crude |
| S4 C2 lung-linked PS/SS | C2 vs C1 | 19/371 vs 113/144 | 647 | 0.065 (0.039–0.110) | 19 | Crude |
| S4 C2 lung-linked PS/SS | C3 vs C1 | 8/602 vs 113/144 | 867 | 0.017 (0.008–0.035) | 8 | Crude |
| S4 C2 lung-linked PS/SS | C2 vs C3 | 19/371 vs 8/602 | 1000 | 3.854 (1.670–8.892) | 8 | Crude |
| S5 HCP only | C2 vs C1 | 34/391 vs 87/115 | 627 | 0.115 (0.073–0.180) | 34 | Crude |
| S5 HCP only | C3 vs C1 | 5/333 vs 87/115 | 540 | 0.020 (0.008–0.050) | 5 | Crude |
| S5 HCP only | C2 vs C3 | 34/391 vs 5/333 | 763 | 5.791 (2.240–14.976) | 5 | Crude |
| S6 exclude literature reports | C2 vs C1 | 25/392 vs 87/127 | 631 | 0.093 (0.057–0.152) | 25 | Crude |
| S6 exclude literature reports | C3 vs C1 | 3/497 vs 87/127 | 714 | 0.009 (0.003–0.028) | 3 | Crude |
| S6 exclude literature reports | C2 vs C3 | 25/392 vs 3/497 | 917 | 10.565 (3.167–35.250) | 3 | Crude |
| S8 NSCLC only | C2 vs C1 | 32/252 vs 98/84 | 466 | 0.109 (0.068–0.174) | 32 | Crude |
| S8 NSCLC only | C3 vs C1 | 6/324 vs 98/84 | 512 | 0.016 (0.007–0.037) | 6 | Crude |
| S8 NSCLC only | C2 vs C3 | 32/252 vs 6/324 | 614 | 6.857 (2.823–16.654) | 6 | Crude |
| S9 SCLC only | C2 vs C1 | 5/163 vs 7/19 | 194 | 0.083 (0.024–0.288) | 5 | Crude |
| S9 SCLC only | C3 vs C1 | 0/94 vs 7/19 | 120 | 0.014 (0.001–0.251) | 0 | Crude |
| S9 SCLC only | C2 vs C3 | 5/163 vs 0/94 | 262 | 6.358 (0.348–116.263) | 0 | Crude |
| S12 exclude anti-VEGF | C2 vs C1 | 33/385 vs 113/141 | 672 | 0.107 (0.069–0.165) | 33 | Crude |
| S12 exclude anti-VEGF | C3 vs C1 | 8/562 vs 113/141 | 824 | 0.018 (0.008–0.037) | 8 | Crude |
| S12 exclude anti-VEGF | C2 vs C3 | 33/385 vs 8/562 | 988 | 6.021 (2.751–13.178) | 8 | Crude |
| **Exploratory Leave One Out** | | | | | | |
| S10 leave out pembrolizumab | C2 vs C1 | 22/329 vs 73/110 | 534 | 0.101 (0.060–0.170) | 22 | Crude |
| S10 leave out pembrolizumab | C3 vs C1 | 8/602 vs 73/110 | 793 | 0.020 (0.009–0.043) | 8 | Crude |
| S10 leave out pembrolizumab | C2 vs C3 | 22/329 vs 8/602 | 961 | 5.032 (2.216–11.428) | 8 | Crude |
| S10 leave out nivolumab | C2 vs C1 | 31/399 vs 66/92 | 588 | 0.108 (0.067–0.176) | 31 | Crude |
| S10 leave out nivolumab | C3 vs C1 | 8/602 vs 66/92 | 768 | 0.019 (0.009–0.040) | 8 | Crude |
| S10 leave out nivolumab | C2 vs C3 | 31/399 vs 8/602 | 1040 | 5.846 (2.660–12.850) | 8 | Crude |
| S10 leave out atezolizumab | C2 vs C1 | 31/242 vs 103/84 | 460 | 0.104 (0.065–0.167) | 31 | Crude |
| S10 leave out atezolizumab | C3 vs C1 | 8/602 vs 103/84 | 797 | 0.011 (0.005–0.023) | 8 | Crude |
| S10 leave out atezolizumab | C2 vs C3 | 31/242 vs 8/602 | 883 | 9.639 (4.369–21.270) | 8 | Crude |
| S10 leave out durvalumab | C2 vs C1 | 34/362 vs 97/125 | 618 | 0.121 (0.078–0.188) | 34 | Crude |
| S10 leave out durvalumab | C3 vs C1 | 8/602 vs 97/125 | 832 | 0.017 (0.008–0.036) | 8 | Crude |
| S10 leave out durvalumab | C2 vs C3 | 34/362 vs 8/602 | 1006 | 7.068 (3.236–15.436) | 8 | Crude |
| S10 leave out cemiplimab | C2 vs C1 | 40/450 vs 113/144 | 747 | 0.113 (0.075–0.170) | 40 | Crude |
| S10 leave out cemiplimab | C3 vs C1 | 8/602 vs 113/144 | 867 | 0.017 (0.008–0.035) | 8 | Crude |
| S10 leave out cemiplimab | C2 vs C3 | 40/450 vs 8/602 | 1100 | 6.689 (3.101–14.430) | 8 | Crude |
| S10 leave out ipilimumab | C2 vs C1 | 36/417 vs 102/132 | 687 | 0.112 (0.073–0.171) | 36 | Crude |
| S10 leave out ipilimumab | C3 vs C1 | 8/602 vs 102/132 | 844 | 0.017 (0.008–0.036) | 8 | Crude |
| S10 leave out ipilimumab | C2 vs C3 | 36/417 vs 8/602 | 1063 | 6.496 (2.989–14.118) | 8 | Crude |
| S10 leave out tremelimumab | C2 vs C1 | 37/438 vs 112/144 | 731 | 0.109 (0.072–0.165) | 37 | Crude |
| S10 leave out tremelimumab | C3 vs C1 | 8/602 vs 112/144 | 866 | 0.017 (0.008–0.036) | 8 | Crude |
| S10 leave out tremelimumab | C2 vs C3 | 37/438 vs 8/602 | 1085 | 6.357 (2.931–13.785) | 8 | Crude |
| S11 leave out era 2015-2017 | C2 vs C1 | 40/449 vs 96/129 | 714 | 0.120 (0.079–0.182) | 40 | Crude |
| S11 leave out era 2015-2017 | C3 vs C1 | 8/428 vs 96/129 | 661 | 0.025 (0.012–0.053) | 8 | Crude |
| S11 leave out era 2015-2017 | C2 vs C3 | 40/449 vs 8/428 | 925 | 4.766 (2.206–10.300) | 8 | Crude |
| S11 leave out era 2018-2020 | C2 vs C1 | 32/377 vs 68/101 | 578 | 0.126 (0.078–0.203) | 32 | Crude |
| S11 leave out era 2018-2020 | C3 vs C1 | 5/411 vs 68/101 | 585 | 0.018 (0.007–0.046) | 5 | Crude |
| S11 leave out era 2018-2020 | C2 vs C3 | 32/377 vs 5/411 | 825 | 6.977 (2.691–18.092) | 5 | Crude |
| S11 leave out era 2021-2023 | C2 vs C1 | 23/221 vs 79/118 | 441 | 0.155 (0.093–0.260) | 23 | Crude |
| S11 leave out era 2021-2023 | C3 vs C1 | 5/495 vs 79/118 | 697 | 0.015 (0.006–0.038) | 5 | Crude |
| S11 leave out era 2021-2023 | C2 vs C3 | 23/221 vs 5/495 | 744 | 10.303 (3.867–27.454) | 5 | Crude |
| S11 leave out era 2024-2026 | C2 vs C1 | 25/318 vs 96/84 | 523 | 0.069 (0.042–0.114) | 25 | Crude |
| S11 leave out era 2024-2026 | C3 vs C1 | 6/472 vs 96/84 | 658 | 0.011 (0.005–0.026) | 6 | Crude |
| S11 leave out era 2024-2026 | C2 vs C3 | 25/318 vs 6/472 | 821 | 6.184 (2.509–15.246) | 6 | Crude |
| **Adjusted role and full-fingerprint sensitivity analyses** | | | | | | |
| S1 ICI role PS+SS | C2 vs C1 | 79/774 vs 114/159 | 1736 | 0.145 (0.099–0.210) | 79 | Firth adjusted |
| S7 full all-DRUG/PT fingerprint dedup | C2 vs C1 | — | 1317 | 0.111 (0.070–0.174) | — | Firth adjusted |

*The primary estimand is the odds of an immune-like rather than marrow-like report phenotype. Sparse leave-one-out cells are retained for transparency but are interpreted descriptively.*

**TABLE S6 Quantitative phenotype-misclassification and adversarial tipping-point analyses.**

| **Database** | **Scenario** | **C2 Se/Sp or move** | **C1 Se/Sp or move** | **Corrected OR** | **Feasible** | **Interpretation** |
| --- | --- | --- | --- | --- | --- | --- |
| **Quantitative bias analysis** | | | | | | |
| FAERS | Perfect classification | 1.00/1.00 | 1.00/1.00 | 0.112 | Yes | Direction retained (OR < 1) |
| FAERS | Nondifferential 5% symmetric error | 0.95/0.95 | 0.95/0.95 | 0.046 | Yes | Direction retained (OR < 1) |
| FAERS | Nondifferential: Se 95%, Sp 99% | 0.95/0.99 | 0.95/0.99 | 0.097 | Yes | Direction retained (OR < 1) |
| FAERS | Nondifferential: Se 90%, Sp 99% | 0.90/0.99 | 0.90/0.99 | 0.093 | Yes | Direction retained (OR < 1) |
| FAERS | Nondifferential: Se 80%, Sp 99% | 0.80/0.99 | 0.80/0.99 | 0.083 | Yes | Direction retained (OR < 1) |
| FAERS | Differential adverse 5% | 0.95/0.99 | 0.99/0.95 | 0.115 | Yes | Direction retained (OR < 1) |
| FAERS | Differential adverse 10% | 0.90/0.99 | 0.99/0.90 | 0.140 | Yes | Direction retained (OR < 1) |
| FAERS | Differential adverse 20% | 0.80/0.99 | 0.99/0.80 | 0.226 | Yes | Direction retained (OR < 1) |
| JADER | Perfect classification | 1.00/1.00 | 1.00/1.00 | 0.055 | Yes | Direction retained (OR < 1) |
| JADER | Nondifferential 5% symmetric error | 0.95/0.95 | 0.95/0.95 | 0.033 | Yes | Direction retained (OR < 1) |
| JADER | Nondifferential: Se 95%, Sp 99% | 0.95/0.99 | 0.95/0.99 | 0.043 | Yes | Direction retained (OR < 1) |
| JADER | Nondifferential: Se 90%, Sp 99% | 0.90/0.99 | 0.90/0.99 | 0.033 | Yes | Direction retained (OR < 1) |
| JADER | Nondifferential: Se 80%, Sp 99% | 0.80/0.99 | 0.80/0.99 | 0.008 | Yes | Direction retained (OR < 1) |
| JADER | Differential adverse 5% | 0.95/0.99 | 0.99/0.95 | 0.056 | Yes | Direction retained (OR < 1) |
| JADER | Differential adverse 10% | 0.90/0.99 | 0.99/0.90 | 0.064 | Yes | Direction retained (OR < 1) |
| JADER | Differential adverse 20% | 0.80/0.99 | 0.99/0.80 | 0.087 | Yes | Direction retained (OR < 1) |
| **Adversarial lower-bound tipping-point analysis** | | | | | | |
| FAERS | Minimum directed relabeling to OR ≥1 | C2 marrow→immune: 1 | C1 immune→marrow: 92 | 1.015 | Yes | 93 reports; 12.4% of all anchors; 35.8% of C1 anchors |
| JADER | Minimum directed relabeling to OR ≥1 | C2 marrow→immune: 0 | C1 immune→marrow: 85 | 1.002 | Yes | 85 reports; 22.6% of all anchors; 61.6% of C1 anchors |

***Note.*** *Se, sensitivity; Sp, specificity. The tipping analysis is an adversarial stress test, not an estimated error rate.*

**TABLE S7
Completeness, distribution, missingness model, and inverse-probability-weighted sensitivity for the report-level onset proxy.**

| **Analysis** | **Group/term** | **N or valid/total** | **Estimate (95% CI or IQR)** | **Raw P** | **Holm P** | **Detail** |
| --- | --- | --- | --- | --- | --- | --- |
| **Onset-proxy completeness** | | | | | | |
| Completeness | C2 marrow-like | 269/455 | 59.1% (54.4–63.7) | — | — | Exact binomial CI |
| Completeness | C1 immune-like | 59/113 | 52.2% (42.6–61.7) | — | — | Exact binomial CI |
| Completeness | C1 marrow-like | 50/144 | 34.7% (27.0–43.1) | — | — | Exact binomial CI |
| Completeness | C2 immune-like | 25/40 | 62.5% (45.8–77.3) | — | — | Exact binomial CI |
| Completeness | C1+C2 marrow-like | 319/599 | 53.3% (49.2–57.3) | — | — | Exact binomial CI |
| Completeness | C1+C2 immune-like | 84/153 | 54.9% (46.7–62.9) | — | — | Exact binomial CI |
| **Onset-proxy distribution by context and phenotype** | | | | | | |
| Distribution | C2 marrow-like | 269 | 9 (5–15) days | — | — | 0–30: 221; 31–90: 36; 91–180: 7; >180: 5 |
| Distribution | C1 immune-like | 59 | 25 (12–66.5) days | — | — | 0–30: 34; 31–90: 12; 91–180: 8; >180: 5 |
| Distribution | C1 marrow-like | 50 | 20 (11–52.25) days | — | — | 0–30: 31; 31–90: 10; 91–180: 6; >180: 3 |
| Distribution | C2 immune-like | 25 | 28 (11–113) days | — | — | 0–30: 13; 31–90: 5; 91–180: 2; >180: 5 |
| Distribution | C1+C2 marrow-like | 319 | 10 (6–21.5) days | — | — | 0–30: 252; 31–90: 46; 91–180: 13; >180: 8 |
| Distribution | C1+C2 immune-like | 84 | 27.5 (11–81.75) days | — | — | 0–30: 47; 31–90: 17; 91–180: 10; >180: 10 |
| **Firth model for onset-proxy availability** | | | | | | |
| Missingness model | (Intercept) | — | 0.157 (0.036–0.667) | 0.012 | — | Adjustment covariate |
| Missingness model | eti_immune | — | 1.314 (0.701–2.465) | 0.394 | 0.394 | Target term |
| Missingness model | treatment_contextC2 | — | 4.552 (2.700–7.808) | <0.001 | <0.001 | Target term |
| Missingness model | report_year_centered | — | 0.805 (0.737–0.877) | <0.001 | — | Adjustment covariate |
| Missingness model | age_imputed | — | 0.992 (0.974–1.011) | 0.410 | — | Adjustment covariate |
| Missingness model | age_missing | — | 0.262 (0.133–0.504) | <0.001 | — | Adjustment covariate |
| Missingness model | sex_modelM | — | 1.134 (0.749–1.714) | 0.551 | — | Adjustment covariate |
| Missingness model | sex_modelUNKNOWN | — | 0.216 (0.095–0.472) | <0.001 | — | Adjustment covariate |
| Missingness model | reporter_groupHCP | — | 6.445 (3.383–12.545) | <0.001 | — | Adjustment covariate |
| Missingness model | reporter_groupOTHER | — | 4.272 (1.541–12.466) | 0.005 | — | Adjustment covariate |
| Missingness model | reporter_groupUNKNOWN | — | 5.182 (1.129–22.652) | 0.035 | — | Adjustment covariate |
| Missingness model | lung_subtypeOTHER_PRIMARY | — | 2.374 (1.329–4.345) | 0.003 | — | Adjustment covariate |
| Missingness model | lung_subtypeSCLC | — | 0.559 (0.354–0.880) | 0.012 | — | Adjustment covariate |
| Missingness model | death | — | 1.120 (0.705–1.795) | 0.633 | — | Adjustment covariate |
| Missingness model | life_threatening | — | 0.716 (0.461–1.114) | 0.138 | — | Adjustment covariate |
| Missingness model | hospitalization | — | 3.875 (2.691–5.622) | <0.001 | — | Adjustment covariate |
| Missingness model | eti_immune:treatment_contextC2 | — | 0.499 (0.191–1.323) | 0.161 | 0.322 | Target term |
| **Inverse-probability-weighted sensitivity** | | | | | | |
| IPW | marrow | 319 | Unweighted 10; IPW 10 days | — | — | Effective N 251.3 |
| IPW | immune | 84 | Unweighted 27.5; IPW 42 days | — | — | Effective N 69.1 |
| IPW | immune minus marrow | 403 | Unweighted 17.5; IPW 32 days | — | — | Difference |

*The IPW analysis used an availability model with weights truncated at the 1st and 99th percentiles and relies on conditional missing-at-random assumptions.*

**TABLE S8 Prespecified evidence modules and their phenotype associations in FAERS and JADER.**

| **Module** | **Clinical evidence domain** | **FAERS immune-like** | **FAERS marrow-like** | **FAERS OR (95% CI)** | **JADER immune-like** | **JADER marrow-like** | **JADER OR (95% CI)** |
| --- | --- | --- | --- | --- | --- | --- | --- |
| B01 | Systemic inflammation/fever | 8 (5.2%) | 50 (8.3%) | 0.636 (0.300–1.345) | 4 (2.8%) | 12 (5.2%) | 0.558 (0.186–1.674) |
| B02 | Pulmonary infection | 14 (9.2%) | 58 (9.7%) | 0.962 (0.526–1.761) | 8 (5.5%) | 21 (9.1%) | 0.605 (0.266–1.378) |
| B03 | Non-pulmonary/systemic infection | 21 (13.7%) | 158 (26.4%) | 0.452 (0.277–0.738) | 21 (14.5%) | 46 (19.9%) | 0.689 (0.394–1.205) |
| B04 | Bleeding/hemorrhagic manifestations | 13 (8.5%) | 53 (8.8%) | 0.982 (0.525–1.834) | 7 (4.8%) | 12 (5.2%) | 0.951 (0.375–2.410) |
| B05 | Thrombotic/embolic manifestations | 4 (2.6%) | 20 (3.3%) | 0.851 (0.302–2.398) | 2 (1.4%) | 5 (2.2%) | 0.717 (0.159–3.246) |
| B06 | Shock/MODS/perfusion failure | 3 (2.0%) | 25 (4.2%) | 0.524 (0.169–1.624) | 0 (0.0%) | 4 (1.7%) | 0.174 (0.009–3.251) |
| B07 | Hepatic injury | 11 (7.2%) | 106 (17.7%) | 0.374 (0.198–0.707) | 6 (4.1%) | 22 (9.5%) | 0.434 (0.177–1.066) |
| B08 | Renal injury | 2 (1.3%) | 81 (13.5%) | 0.105 (0.029–0.374) | 0 (0.0%) | 20 (8.7%) | 0.035 (0.002–0.591) |
| B09 | GI/mucosal inflammation/toxicity | 9 (5.9%) | 177 (29.5%) | 0.156 (0.079–0.309) | 3 (2.1%) | 29 (12.6%) | 0.169 (0.055–0.521) |
| B10 | Noninfectious respiratory injury/failure | 14 (9.2%) | 106 (17.7%) | 0.482 (0.270–0.860) | 14 (9.7%) | 22 (9.5%) | 1.027 (0.512–2.058) |
| B11 | Cardiovascular injury/arrhythmia | 4 (2.6%) | 34 (5.7%) | 0.493 (0.182–1.339) | 1 (0.7%) | 6 (2.6%) | 0.360 (0.060–2.152) |
| B12 | Neurologic/encephalopathic/neuromuscular | 5 (3.3%) | 62 (10.4%) | 0.319 (0.131–0.776) | 1 (0.7%) | 5 (2.2%) | 0.427 (0.069–2.632) |
| B13 | Endocrine/metabolic/electrolyte | 10 (6.5%) | 142 (23.7%) | 0.235 (0.122–0.452) | 7 (4.8%) | 34 (14.7%) | 0.310 (0.137–0.703) |
| B14 | Dermatologic/musculoskeletal | 15 (9.8%) | 114 (19.0%) | 0.475 (0.270–0.833) | 5 (3.4%) | 29 (12.6%) | 0.269 (0.105–0.685) |
| B15 | Other explicit immune-mediated/autoimmune irAE | 23 (15.0%) | 33 (5.5%) | 3.045 (1.738–5.335) | 14 (9.7%) | 18 (7.8%) | 1.273 (0.619–2.617) |
| B16 | Constitutional/general deterioration | 3 (2.0%) | 131 (21.9%) | 0.083 (0.028–0.243) | 2 (1.4%) | 17 (7.4%) | 0.214 (0.056–0.817) |

*Values are module-present n (%) unless otherwise indicated. ORs compare module presence in immune-like versus marrow-like anchors and use Haldane correction; they are descriptive, not independent causal effects.*

**TABLE S9 Model specification and nested cross-validation results.**

| **Component** | **Model/fold** | **Training N** | **Validation N** | **C** | **l1 ratio** | **AUROC** | **PR-AUC** | **Brier** | **Log-loss** | **Details** |
| --- | --- | --- | --- | --- | --- | --- | --- | --- | --- | --- |
| **Model specification** | | | | | | | | | | |
| Predictors | Primary | — | — | — | — | — | — | — | — | age_years; report_year; sex_model; reporter_group; treatment_context; ici_class_combo; dual_ici; anti_vegf; chemo_platinum; chemo_pemetrexed; chemo_taxane; chemo_topoisomerase; chemo_other |
| Predictors | Secondary | — | — | — | — | — | — | — | — | Primary features plus B01–B16 and module_zero |
| Preprocessing | Both | — | — | — | — | — | — | — | — | Numeric: median imputation with missingness indicators, then standardization. Categorical: most-frequent imputation and one-hot encoding with unknown levels ignored. Binary: constant-zero imputation. All preprocessing was fitted within each training fold. |
| Final classifier | Both | 604 | — | 0.3 | 0.25 | — | — | — | — | Elastic-net logistic regression; solver=saga; penalty=elasticnet; class_weight=None; max_iter=20000; tolerance=1×10⁻⁴; seed 20260815 |
| Nested CV | Both | — | — | 0.01, 0.03, 0.1, 0.3, 1, 3, 10 | 0, 0.25, 0.5, 0.75, 1 | — | — | — | — | 5 stratified outer folds × 4 stratified inner folds; inner objective, negative log-loss; outer seed 20260815; inner seed 20260815 plus outer-fold number |
| **Outer-fold validation metrics** | | | | | | | | | | |
| Outer fold | Primary, fold 1 | 483 | 121 | 0.3 | 0 | 0.879 | 0.757 | 0.101 | 0.345 | Cal. intercept 0.267; slope 1.439 |
| Outer fold | Secondary, fold 1 | 483 | 121 | 0.3 | 0 | 0.900 | 0.796 | 0.094 | 0.316 | Cal. intercept 0.145; slope 1.338 |
| Outer fold | Primary, fold 2 | 483 | 121 | 0.3 | 0 | 0.874 | 0.679 | 0.121 | 0.371 | Cal. intercept 0.703; slope 1.297 |
| Outer fold | Secondary, fold 2 | 483 | 121 | 0.3 | 0 | 0.881 | 0.695 | 0.115 | 0.356 | Cal. intercept 0.659; slope 1.197 |
| Outer fold | Primary, fold 3 | 483 | 121 | 0.3 | 0.25 | 0.806 | 0.608 | 0.130 | 0.416 | Cal. intercept -0.100; slope 0.886 |
| Outer fold | Secondary, fold 3 | 483 | 121 | 0.3 | 0 | 0.833 | 0.649 | 0.120 | 0.390 | Cal. intercept -0.082; slope 0.960 |
| Outer fold | Primary, fold 4 | 483 | 121 | 0.3 | 0 | 0.817 | 0.523 | 0.137 | 0.411 | Cal. intercept -0.147; slope 0.939 |
| Outer fold | Secondary, fold 4 | 483 | 121 | 1 | 0.75 | 0.838 | 0.568 | 0.132 | 0.389 | Cal. intercept -0.198; slope 0.862 |
| Outer fold | Primary, fold 5 | 484 | 120 | 0.3 | 0.25 | 0.804 | 0.510 | 0.138 | 0.425 | Cal. intercept -0.245; slope 0.859 |
| Outer fold | Secondary, fold 5 | 484 | 120 | 1 | 0.5 | 0.854 | 0.623 | 0.123 | 0.376 | Cal. intercept -0.174; slope 0.887 |
| **Locked thresholds and operating characteristics** | | | | | | | | | | |
| Operating point | FAERS development OOF; Primary | — | 604 | — | — | — | — | — | 0.393 | Locked threshold 0.213; sensitivity 0.789; specificity 0.749 |
| Operating point | FAERS development OOF; Secondary | — | 604 | — | — | — | — | — | 0.365 | Locked threshold 0.207; sensitivity 0.812; specificity 0.749 |
| Operating point | FAERS temporal validation; Primary | — | 148 | — | — | — | — | — | 0.343 | Locked threshold 0.213; sensitivity 0.600; specificity 0.789 |
| Operating point | FAERS temporal validation; Secondary | — | 148 | — | — | — | — | — | 0.317 | Locked threshold 0.207; sensitivity 0.750; specificity 0.750 |
| Operating point | JADER cross-database transport; Primary | — | 376 | — | — | — | — | — | 0.489 | Locked threshold 0.213; sensitivity 0.779; specificity 0.861 |
| Operating point | JADER cross-database transport; Secondary | — | 376 | — | — | — | — | — | 0.449 | Locked threshold 0.207; sensitivity 0.841; specificity 0.805 |

*Hyperparameters shown for the final models were fixed before the temporal evaluation and cross-database transport evaluation. Out-of-fold predictions were used for development performance; temporal and JADER transport predictions used the locked final report-phenotype model. No class weighting or synthetic oversampling was used.*

**TABLE S10 Quintile calibration in FAERS temporal evaluation and JADER cross-database transport evaluation.**

| **Model** | **Bin** | **N** | **Immune-like events** | **Mean predicted probability** | **Observed proportion (95% CI)** | **Binning** | **Interval method** |
| --- | --- | --- | --- | --- | --- | --- | --- |
| **FAERS temporal validation** | | | | | | | |
| Primary | 1 | 30 | 0 | 0.017 | 0.000 (0.000–0.116) | equal-frequency quintiles | Clopper-Pearson 95% CI |
| Primary | 2 | 29 | 4 | 0.062 | 0.138 (0.039–0.317) | equal-frequency quintiles | Clopper-Pearson 95% CI |
| Primary | 3 | 30 | 2 | 0.128 | 0.067 (0.008–0.221) | equal-frequency quintiles | Clopper-Pearson 95% CI |
| Primary | 4 | 29 | 5 | 0.194 | 0.172 (0.058–0.358) | equal-frequency quintiles | Clopper-Pearson 95% CI |
| Primary | 5 | 30 | 9 | 0.414 | 0.300 (0.147–0.494) | equal-frequency quintiles | Clopper-Pearson 95% CI |
| Secondary | 1 | 30 | 0 | 0.008 | 0.000 (0.000–0.116) | equal-frequency quintiles | Clopper-Pearson 95% CI |
| Secondary | 2 | 29 | 1 | 0.042 | 0.034 (0.001–0.178) | equal-frequency quintiles | Clopper-Pearson 95% CI |
| Secondary | 3 | 30 | 3 | 0.117 | 0.100 (0.021–0.265) | equal-frequency quintiles | Clopper-Pearson 95% CI |
| Secondary | 4 | 29 | 4 | 0.219 | 0.138 (0.039–0.317) | equal-frequency quintiles | Clopper-Pearson 95% CI |
| Secondary | 5 | 30 | 12 | 0.460 | 0.400 (0.227–0.594) | equal-frequency quintiles | Clopper-Pearson 95% CI |
| **JADER cross-database transport evaluation** | | | | | | | |
| Primary | 1 | 76 | 4 | 0.034 | 0.053 (0.015–0.129) | equal-frequency quintiles | Clopper-Pearson 95% CI |
| Primary | 2 | 75 | 7 | 0.077 | 0.093 (0.038–0.183) | equal-frequency quintiles | Clopper-Pearson 95% CI |
| Primary | 3 | 75 | 19 | 0.145 | 0.253 (0.160–0.367) | equal-frequency quintiles | Clopper-Pearson 95% CI |
| Primary | 4 | 75 | 49 | 0.368 | 0.653 (0.535–0.760) | equal-frequency quintiles | Clopper-Pearson 95% CI |
| Primary | 5 | 75 | 66 | 0.633 | 0.880 (0.784–0.944) | equal-frequency quintiles | Clopper-Pearson 95% CI |
| Secondary | 1 | 76 | 2 | 0.036 | 0.026 (0.003–0.092) | equal-frequency quintiles | Clopper-Pearson 95% CI |
| Secondary | 2 | 75 | 7 | 0.087 | 0.093 (0.038–0.183) | equal-frequency quintiles | Clopper-Pearson 95% CI |
| Secondary | 3 | 75 | 21 | 0.178 | 0.280 (0.182–0.396) | equal-frequency quintiles | Clopper-Pearson 95% CI |
| Secondary | 4 | 75 | 50 | 0.412 | 0.667 (0.548–0.771) | equal-frequency quintiles | Clopper-Pearson 95% CI |
| Secondary | 5 | 75 | 65 | 0.684 | 0.867 (0.768–0.934) | equal-frequency quintiles | Clopper-Pearson 95% CI |

*Each model–dataset combination used five equal-frequency bins. Observed intervals are exact Clopper–Pearson 95% confidence intervals.*

**TABLE S11 Report-score results under prespecified model and threshold analyses.**

| **Analysis** | **Model** | **Thresholds** | **Subset** | **Subset N** | **Classification** | **n** | **%** |
| --- | --- | --- | --- | --- | --- | --- | --- |
| **Prespecified thresholds** | | | | | | | |
| Prespecified thresholds | Secondary | 0.30/0.70 | All undifferentiated C1+C2 reports | 927 | High immune-like score | 43 | 4.6% |
| Prespecified thresholds | Secondary | 0.30/0.70 | All undifferentiated C1+C2 reports | 927 | Intermediate score | 449 | 48.4% |
| Prespecified thresholds | Secondary | 0.30/0.70 | All undifferentiated C1+C2 reports | 927 | Low immune-like score | 435 | 46.9% |
| Prespecified thresholds | Primary | 0.30/0.70 | All undifferentiated C1+C2 reports | 927 | High immune-like score | 39 | 4.2% |
| Prespecified thresholds | Primary | 0.30/0.70 | All undifferentiated C1+C2 reports | 927 | Intermediate score | 450 | 48.5% |
| Prespecified thresholds | Primary | 0.30/0.70 | All undifferentiated C1+C2 reports | 927 | Low immune-like score | 438 | 47.2% |
| **Threshold sensitivity** | | | | | | | |
| Threshold sensitivity | Secondary | 0.40/0.60 | All undifferentiated C1+C2 reports | 927 | High immune-like score | 142 | 15.3% |
| Threshold sensitivity | Secondary | 0.40/0.60 | All undifferentiated C1+C2 reports | 927 | Intermediate score | 241 | 26.0% |
| Threshold sensitivity | Secondary | 0.40/0.60 | All undifferentiated C1+C2 reports | 927 | Low immune-like score | 544 | 58.7% |
| Threshold sensitivity | Secondary | 0.20/0.80 | All undifferentiated C1+C2 reports | 927 | High immune-like score | 4 | 0.4% |
| Threshold sensitivity | Secondary | 0.20/0.80 | All undifferentiated C1+C2 reports | 927 | Intermediate score | 588 | 63.4% |
| Threshold sensitivity | Secondary | 0.20/0.80 | All undifferentiated C1+C2 reports | 927 | Low immune-like score | 335 | 36.1% |
| **Evidence-module restriction** | | | | | | | |
| Evidence-module restriction | Secondary | 0.30/0.70 | Reports with ≥1 non-zero evidence module | 484 | High immune-like score | 17 | 3.5% |
| Evidence-module restriction | Secondary | 0.30/0.70 | Reports with ≥1 non-zero evidence module | 484 | Intermediate score | 202 | 41.7% |
| Evidence-module restriction | Secondary | 0.30/0.70 | Reports with ≥1 non-zero evidence module | 484 | Low immune-like score | 265 | 54.8% |

*Threshold notation is low/high immune-like score. The evidence-module restriction includes only reports with at least one non-zero evidence module. Categories describe report-phenotype scores, not confirmed diagnoses.*

**TABLE S12 Exploratory heterogeneity of the C2-versus-C1 report-phenotype composition association.**

| **Stratum** | **Immune/marrow cells** | **N** | **Minimum cell** | **Estimate type** | **OR (95% CI)** | **P value** | **BH q** | **Inference status** |
| --- | --- | --- | --- | --- | --- | --- | --- | --- |
| **ICI agent** | | | | | | | | |
| pembrolizumab | 18/126 vs 40/34 | 218 | 18 | Adjusted | 0.131 (0.059–0.275) | <0.001 | <0.001 | Inferential (minimum cell >=5) |
| nivolumab | 9/56 vs 47/52 | 164 | 9 | Adjusted | 0.154 (0.060–0.360) | <0.001 | <0.001 | Inferential (minimum cell >=5) |
| atezolizumab | 9/213 vs 10/60 | 292 | 9 | Adjusted | 0.063 (0.015–0.228) | <0.001 | <0.001 | Inferential (minimum cell >=5) |
| durvalumab | 6/93 vs 16/19 | 134 | 6 | Adjusted | 0.139 (0.038–0.446) | <0.001 | 0.001 | Inferential (minimum cell >=5) |
| cemiplimab | 0/5 vs 0/0 | 5 | 0 | Crude | 0.091 (0.001–11.885) | 1.000 | 1.000 | Descriptive only (minimum cell <5) |
| ipilimumab | 4/38 vs 11/12 | 65 | 4 | Crude | 0.115 (0.031–0.428) | 0.001 | 0.002 | Descriptive only (minimum cell <5) |
| tremelimumab | 3/17 vs 1/0 | 21 | 0 | Crude | 0.067 (0.002–1.998) | 0.190 | 0.222 | Descriptive only (minimum cell <5) |
| **Chemotherapy class** | | | | | | | | |
| platinum | 37/427 vs 113/144 | 721 | 37 | Adjusted | 0.109 (0.067–0.172) | <0.001 | <0.001 | Inferential (minimum cell >=5) |
| pemetrexed | 12/137 vs 113/144 | 406 | 12 | Adjusted | 0.094 (0.046–0.178) | <0.001 | <0.001 | Inferential (minimum cell >=5) |
| taxane | 21/135 vs 113/144 | 413 | 21 | Adjusted | 0.179 (0.100–0.311) | <0.001 | <0.001 | Inferential (minimum cell >=5) |
| topoisomerase | 4/177 vs 113/144 | 438 | 4 | Crude | 0.029 (0.010–0.080) | <0.001 | <0.001 | Descriptive only (minimum cell <5) |
| other | 3/13 vs 113/144 | 273 | 3 | Crude | 0.294 (0.082–1.057) | 0.066 | 0.066 | Descriptive only (minimum cell <5) |
| **Reporter country** | | | | | | | | |
| JP | 32/237 vs 65/41 | 375 | 32 | Adjusted | 0.084 (0.043–0.157) | <0.001 | <0.001 | Inferential (minimum cell >=5) |
| CN | 2/55 vs 4/19 | 80 | 2 | Crude | 0.173 (0.029–1.020) | 0.054 | 0.107 | Descriptive only (minimum cell <5) |
| US | 2/27 vs 14/11 | 54 | 2 | Crude | 0.058 (0.011–0.300) | <0.001 | <0.001 | Descriptive only (minimum cell <5) |
| FR | 3/15 vs 5/18 | 41 | 3 | Crude | 0.720 (0.147–3.520) | 1.000 | 1.000 | Descriptive only (minimum cell <5) |
| EU | 0/25 vs 1/3 | 29 | 0 | Crude | 0.046 (0.002–1.357) | 0.138 | 0.221 | Descriptive only (minimum cell <5) |
| KR | 0/16 vs 0/9 | 25 | 0 | Crude | 0.576 (0.011–31.449) | 1.000 | 1.000 | Descriptive only (minimum cell <5) |
| DE | 0/13 vs 5/6 | 24 | 0 | Crude | 0.044 (0.002–0.917) | 0.011 | 0.029 | Descriptive only (minimum cell <5) |
| IT | 0/11 vs 2/9 | 22 | 0 | Crude | 0.165 (0.007–3.877) | 0.476 | 0.635 | Descriptive only (minimum cell <5) |

*Adjusted estimates are shown only when all four cells met the prespecified minimum-cell criterion; sparse strata are descriptive. All subgroup analyses are exploratory.*

**TABLE S13 Detailed concordance of the two independent clinical reviews.**

| **Review set** | **Reviewer A category/metric** | **Reviewer B category** | **Count** | **Agreement** | **Summary statistic** |
| --- | --- | --- | --- | --- | --- |
| **Summary agreement** | | | | | |
| 39-report proxy set | Summary | — | 36/39 | 92.3% | κ=0.894; AC1=0.899 |
| DIC-like set | Summary | — | 20/22 | 90.9% | κ=0.879; AC1=0.888 |
| **Non-zero reviewer A-by-reviewer B confusion cells** | | | | | |
| 39-report proxy set | Immune probable | Immune probable | 8 | A-row total 8 | — |
| 39-report proxy set | Immune possible | Immune possible | 14 | A-row total 14 | — |
| 39-report proxy set | Non-immune probable | Non-immune probable | 6 | A-row total 6 | — |
| 39-report proxy set | Indeterminate | Immune possible | 2 | A-row total 11 | — |
| 39-report proxy set | Indeterminate | Non-immune probable | 1 | A-row total 11 | — |
| 39-report proxy set | Indeterminate | Indeterminate | 8 | A-row total 11 | — |
| DIC-like set | Sepsis related | Sepsis related | 5 | A-row total 5 | — |
| DIC-like set | Malignancy related | Malignancy related | 3 | A-row total 3 | — |
| DIC-like set | Treatment-related immune/coagulation | Treatment-related immune/coagulation | 7 | A-row total 7 | — |
| DIC-like set | Multifactorial | Sepsis related | 1 | A-row total 6 | — |
| DIC-like set | Multifactorial | Multifactorial | 5 | A-row total 6 | — |
| DIC-like set | Insufficient information | Malignancy related | 1 | A-row total 1 | — |
| **Category-specific exact agreement** | | | | | |
| 39-report | Immune probable | Same category | 8/8 | 100.0% | — |
| 39-report | Immune possible | Same category | 14/14 | 100.0% | — |
| 39-report | Nonimmune probable | Same category | 6/6 | 100.0% | — |
| 39-report | Indeterminate | Same category | 8/11 | 72.7% | — |
| DIC-like | Sepsis | Same category | 5/5 | 100.0% | — |
| DIC-like | Malignancy | Same category | 3/3 | 100.0% | — |
| DIC-like | Treatment-related immune/coag | Same category | 7/7 | 100.0% | — |
| DIC-like | Multifactorial | Same category | 5/6 | 83.3% | — |
| DIC-like | Insufficient | Same category | 0/1 | 0.0% | — |

Cohen’s κ and Gwet’s AC1 were calculated before third-person adjudication. This legacy enrichment/content review is retained for transparency; the random stratified 120-report review appears in main Figure 8 and Tables S25–S33.

**TABLE S14
Case-level independent review and final consensus for the 39-report model-selected proxy set.**

| **No.** | **PrimaryID** | **Quarter; context** | **Age/sex** | **ICI regimen** | **Primary/secondary probability** | **Key structured evidence** | **Reviewer A / B** | **Final consensus** | **Confidence** | **Decision rationale** |
| --- | --- | --- | --- | --- | --- | --- | --- | --- | --- | --- |
| 1 | 239389111 | 2024q2  C1 | 84/M | nivolumab | 0.730 /  0.871 | Modules: B04, B05, B15; Flags: bleeding, other immune-related AE; Key PTs: Autoimmune haemolytic anaemia; Thrombotic thrombocytopenic purpura | Immune probable /  Immune probable | Immune probable | 5/5 | Initial agreement; explicit immune or autoimmune features supported an immune etiology. |
| 2 | 156987163 | 2019q1  C1 | 108/M | nivolumab | 0.881 /  0.858 | Modules: none; Flags: no automated competing-cause flag | Indeterminate /  Indeterminate | Indeterminate | 2/5 | Initial agreement; isolated thrombocytopenia or insufficient temporal and mechanistic evidence precluded attribution. |
| 3 | 235197146 | 2026q1  C1 | 80/M | nivolumab+  ipilimumab | 0.592 /  0.828 | Modules: B07, B15; Flags: other immune-related AE; Key PTs: Immune-mediated hepatitis | Immune probable /  Immune probable | Immune probable | 4/5 | Initial agreement; explicit immune or autoimmune features supported an immune etiology. |
| 4 | 169961852 | 2019q4  C1 | 83/M | nivolumab+  ipilimumab | 0.725 /  0.807 | Modules: none; Flags: no automated competing-cause flag | Immune possible /  Immune possible | Immune possible | 3/5 | Initial agreement; an immune etiology was plausible, but the structured evidence was incomplete. |
| 5 | 124448711 | 2016q2  C1 | 78/M | nivolumab | 0.784 /  0.800 | Modules: none; Flags: no automated competing-cause flag | Indeterminate /  Immune possible | Indeterminate | 2/5 | Third-person adjudication; isolated thrombocytopenia or insufficient temporal and mechanistic evidence precluded attribution. |
| 6 | 128731611 | 2016q4  C1 | 77/M | nivolumab | 0.777 /  0.795 | Modules: none; Flags: no automated competing-cause flag; Key PTs: Anaemia | Indeterminate /  Immune possible | Indeterminate | 2/5 | Third-person adjudication; isolated thrombocytopenia or insufficient temporal and mechanistic evidence precluded attribution. |
| 7 | 129530149 | 2019q3  C1 | 77/M | nivolumab | 0.764 /  0.795 | Modules: none; Flags: no automated competing-cause flag; Key PTs: Malignant neoplasm progression | Non-immune probable /  Non-immune probable | Non-immune probable | 4/5 | Initial agreement; a stronger non-immune competing explanation was present. |
| 8 | 161278543 | 2019q4  C1 | 77/M | pembrolizumab | 0.764 /  0.795 | Modules: none; Flags: no automated competing-cause flag | Indeterminate /  Indeterminate | Indeterminate | 2/5 | Initial agreement; isolated thrombocytopenia or insufficient temporal and mechanistic evidence precluded attribution. |
| 9 | 151635511 | 2018q3  C1 | 76/M | nivolumab | 0.762 /  0.790 | Modules: none; Flags: no automated competing-cause flag; Key PTs: Malignant neoplasm progression | Non-immune probable /  Non-immune probable | Non-immune probable | 3/5 | Initial agreement; a stronger non-immune competing explanation was present. |
| 10 | 132547673 | 2017q2  C1 | 83/M | nivolumab | 0.809 /  0.769 | Modules: B11; Flags: no automated competing-cause flag | Immune possible /  Immune possible | Immune possible | 3/5 | Initial agreement; an immune etiology was plausible, but the structured evidence was incomplete. |
| 11 | 188399761 | 2021q1  C1 | 87/M | pembrolizumab | 0.765 /  0.767 | Modules: none; Flags: infection/sepsis | Immune possible /  Immune possible | Immune possible | 3/5 | Initial agreement; an immune etiology was plausible, but the structured evidence was incomplete. |
| 12 | 158305545 | 2023q1  C1 | 76/F | pembrolizumab | 0.569 /  0.764 | Modules: B07, B15; Flags: other immune-related AE; Key PTs: Immune-mediated hepatic disorder | Immune probable /  Immune probable | Immune probable | 4/5 | Initial agreement; explicit immune or autoimmune features supported an immune etiology. |
| 13 | 229814721 | 2023q3  C1 | 96/M | pembrolizumab | 0.812 /  0.763 | Modules: B01; Flags: no automated competing-cause flag | Immune possible /  Immune possible | Immune possible | 2/5 | Initial agreement; an immune etiology was plausible, but the structured evidence was incomplete. |
| 14 | 143643642 | 2018q1  C1 | 62/M | nivolumab | 0.656 /  0.759 | Modules: B13, B15; Flags: other immune-related AE; Key PTs: Autoimmune thyroiditis | Immune probable /  Immune probable | Immune probable | 4/5 | Initial agreement; explicit immune or autoimmune features supported an immune etiology. |
| 15 | 130264311 | 2016q4  C1 | 78/M | nivolumab | 0.784 /  0.759 | Modules: B03, B10, B14; Flags: other immune-related AE | Immune probable /  Immune probable | Immune probable | 4/5 | Initial agreement; explicit immune or autoimmune features supported an immune etiology. |
| 16 | 165667622 | 2019q3  C1 | 87/F | pembrolizumab | 0.750 /  0.750 | Modules: B09, B14, B15; Flags: no automated competing-cause flag; Key PTs: Anaemia | Immune probable /  Immune probable | Immune probable | 4/5 | Initial agreement; explicit immune or autoimmune features supported an immune etiology. |
| 17 | 211570222 | 2022q3  C1 | 83/M | pembrolizumab | 0.732 /  0.746 | Modules: none; Flags: no automated competing-cause flag | Immune possible /  Immune possible | Immune possible | 3/5 | Initial agreement; an immune etiology was plausible, but the structured evidence was incomplete. |
| 18 | 155080121 | 2018q4  C1 | 67/M | nivolumab | 0.697 /  0.744 | Modules: none; Flags: no automated competing-cause flag | Indeterminate /  Indeterminate | Indeterminate | 2/5 | Initial agreement; isolated thrombocytopenia or insufficient temporal and mechanistic evidence precluded attribution. |
| 19 | 258241021 | 2025q3  C1 | 79/M | pembrolizumab | 0.579 /  0.742 | Modules: B01, B14, B15; Flags: no automated competing-cause flag | Immune probable /  Immune probable | Immune probable | 4/5 | Initial agreement; explicit immune or autoimmune features supported an immune etiology. |
| 20 | 145692941 | 2018q1  C1 | 70/M | nivolumab+  ipilimumab | 0.626 /  0.741 | Modules: none; Flags: no automated competing-cause flag | Immune possible /  Immune possible | Immune possible | 3/5 | Initial agreement; an immune etiology was plausible, but the structured evidence was incomplete. |
| 21 | 183989482 | 2020q4  C1 | 96/M | pembrolizumab | 0.744 /  0.741 | Modules: none; Flags: no automated competing-cause flag | Indeterminate /  Indeterminate | Indeterminate | 2/5 | Initial agreement; isolated thrombocytopenia or insufficient temporal and mechanistic evidence precluded attribution. |
| 22 | 222441791 | 2023q2  C1 | 81/M | pembrolizumab | 0.713 /  0.734 | Modules: none; Flags: no automated competing-cause flag | Immune possible /  Immune possible | Immune possible | 3/5 | Initial agreement; an immune etiology was plausible, but the structured evidence was incomplete. |
| 23 | 163800863 | 2019q3  C1 | 108/F | pembrolizumab | 0.748 /  0.733 | Modules: none; Flags: no automated competing-cause flag | Indeterminate /  Indeterminate | Indeterminate | 1/5 | Initial agreement; isolated thrombocytopenia or insufficient temporal and mechanistic evidence precluded attribution. |
| 24 | 154355861 | 2018q3  C1 | 68/M | nivolumab+  ipilimumab | 0.609 /  0.730 | Modules: none; Flags: no automated competing-cause flag | Immune possible /  Immune possible | Immune possible | 3/5 | Initial agreement; an immune etiology was plausible, but the structured evidence was incomplete. |
| 25 | 176125911 | 2020q2  C1 | 80/M | pembrolizumab | 0.720 /  0.729 | Modules: none; Flags: no automated competing-cause flag | Immune possible /  Immune possible | Immune possible | 3/5 | Initial agreement; an immune etiology was plausible, but the structured evidence was incomplete. |
| 26 | 113980592 | 2015q4  C1 | 64/M | nivolumab | 0.689 /  0.728 | Modules: none; Flags: no automated competing-cause flag; Key PTs: Malignant neoplasm progression | Non-immune probable /  Non-immune probable | Non-immune probable | 4/5 | Initial agreement; a stronger non-immune competing explanation was present. |
| 27 | 173458821 | 2020q1  C1 | 79/M | pembrolizumab | 0.713 /  0.723 | Modules: none; Flags: no automated competing-cause flag | Indeterminate /  Indeterminate | Indeterminate | 2/5 | Initial agreement; isolated thrombocytopenia or insufficient temporal and mechanistic evidence precluded attribution. |
| 28 | 182611871 | 2020q3  C1 | 79/M | pembrolizumab | 0.713 /  0.723 | Modules: none; Flags: no automated competing-cause flag | Immune possible /  Immune possible | Immune possible | 2/5 | Initial agreement; an immune etiology was plausible, but the structured evidence was incomplete. |
| 29 | 123349312 | 2016q2  C1 | 69/M | nivolumab | 0.722 /  0.723 | Modules: B07; Flags: no automated competing-cause flag; Key PTs: Malignant neoplasm progression | Non-immune probable /  Non-immune probable | Non-immune probable | 4/5 | Initial agreement; a stronger non-immune competing explanation was present. |
| 30 | 145598221 | 2018q1  C1 | 63/M | nivolumab | 0.665 /  0.722 | Modules: none; Flags: no automated competing-cause flag | Immune possible /  Immune possible | Immune possible | 3/5 | Initial agreement; an immune etiology was plausible, but the structured evidence was incomplete. |
| 31 | 157637524 | 2020q1  C1 | 90/M | nivolumab | 0.788 /  0.722 | Modules: B05, B11; Flags: other immune-related AE; Key PTs: Malignant neoplasm progression | Immune possible /  Immune possible | Immune possible | 3/5 | Initial agreement; an immune etiology was plausible, but the structured evidence was incomplete. |
| 32 | 144344091 | 2018q1  C1 | 70/M | ipilimumab | 0.626 /  0.720 | Modules: none; Flags: no automated competing-cause flag | Immune possible /  Immune possible | Immune possible | 3/5 | Initial agreement; an immune etiology was plausible, but the structured evidence was incomplete. |
| 33 | 185140302 | 2021q1  C1 | 78/M | pembrolizumab | 0.700 /  0.717 | Modules: none; Flags: no automated competing-cause flag | Indeterminate /  Indeterminate | Indeterminate | 2/5 | Initial agreement; isolated thrombocytopenia or insufficient temporal and mechanistic evidence precluded attribution. |
| 34 | 163808342 | 2019q2  C1 | 70/M | nivolumab | 0.715 /  0.716 | Modules: B02, B03, B11; Flags: infection/sepsis; Key PTs: Pneumonia; Staphylococcal infection | Non-immune probable /  Non-immune probable | Non-immune probable | 5/5 | Initial agreement; a stronger non-immune competing explanation was present. |
| 35 | 229685701 | 2023q3  C1 | 81/M | pembrolizumab | 0.713 /  0.713 | Modules: B10; Flags: no automated competing-cause flag | Indeterminate /  Non-immune probable | Indeterminate | 3/5 | Third-person adjudication; isolated thrombocytopenia or insufficient temporal and mechanistic evidence precluded attribution. |
| 36 | 126682181 | 2016q3  C1 | 65/M | nivolumab+  ipilimumab | 0.594 /  0.713 | Modules: none; Flags: no automated competing-cause flag | Indeterminate /  Indeterminate | Indeterminate | 2/5 | Initial agreement; isolated thrombocytopenia or insufficient temporal and mechanistic evidence precluded attribution. |
| 37 | 240191141 | 2024q2  C1 | 81/M | durvalumab | 0.489 /  0.712 | Modules: B03, B07, B10, B15; Flags: other immune-related AE; Key PTs: Autoimmune anaemia | Immune probable /  Immune probable | Immune probable | 5/5 | Initial agreement; explicit immune or autoimmune features supported an immune etiology. |
| 38 | 194720521 | 2021q2  C1 | 71/M | pembrolizumab | 0.643 /  0.709 | Modules: B02, B03, B05, B07, B11, B12, B13, B15; Flags: infection/sepsis, other immune-related AE; Key PTs: Immune-mediated hepatitis; Pneumonia; Streptococcal sepsis | Non-immune probable /  Non-immune probable | Non-immune probable | 4/5 | Initial agreement; a stronger non-immune competing explanation was present. |
| 39 | 172092421 | 2019q4  C1 | 76/M | nivolumab | 0.695 /  0.706 | Modules: none; Flags: no automated competing-cause flag | Immune possible /  Immune possible | Immune possible | 3/5 | Initial agreement; an immune etiology was plausible, but the structured evidence was incomplete. |

*The 39-report set was selected by model ranking and is enrichment biased. Probabilities are report-level phenotype scores and not diagnostic probabilities. PrimaryID is the public FAERS report identifier.*

*Age is shown as reported; extreme values were not manually corrected. Key evidence summarizes prespecified modules, automated competing-cause flags, and up to three salient co-reported MedDRA preferred terms.*

**TABLE S15 Case-level independent review and final consensus for the 22-report DIC-like set.**

| **No.** | **PrimaryID** | **Quarter; context** | **Age/sex** | **ICI regimen** | **Key structured evidence** | **Reviewer A / B** | **Final consensus** | **Confidence** | **Decision rationale** |
| --- | --- | --- | --- | --- | --- | --- | --- | --- | --- |
| 1 | 2313107521 | 2024q2  C2 | 60/M | nivolumab+  ipilimumab | Modules: B03, B07, B14; Flags: infection/sepsis, DIC/coagulopathy; Key PTs: Coagulopathy; Haemophagocytic lymphohistiocytosis; Sepsis | Multifactorial /  Multifactorial | Multifactorial | 4/5 | Initial agreement; multiple competing mechanisms precluded a single attribution. |
| 2 | 205053465 | 2023q1  C2 | 61/M | nivolumab+  ipilimumab | Modules: B01, B03, B10; Flags: DIC/coagulopathy; Key PTs: Disseminated intravascular coagulation | Treatment-related immune/coagulation /  Treatment-related immune/coagulation | Treatment-related immune/coagulation | 4/5 | Initial agreement; treatment-related immune or coagulation toxicity was considered most plausible. |
| 3 | 199229191 | 2021q4  C1 | 63/M | atezolizumab | Modules: B03, B07, B09, B10; Flags: DIC/coagulopathy, other immune-related AE; Key PTs: Coagulopathy | Treatment-related immune/coagulation /  Treatment-related immune/coagulation | Treatment-related immune/coagulation | 4/5 | Initial agreement; treatment-related immune or coagulation toxicity was considered most plausible. |
| 4 | 126055067 | 2018q2  C1 | 66/M | nivolumab | Modules: B14, B16; Flags: DIC/coagulopathy; Key PTs: Anaemia; Coagulopathy; Malignant neoplasm progression | Malignancy related /  Malignancy related | Malignancy related | 4/5 | Initial agreement; malignancy or progression was the dominant competing mechanism. |
| 5 | 137925375 | 2017q4  C1 | 64/F | pembrolizumab | Modules: B10; Flags: DIC/coagulopathy; Key PTs: Disseminated intravascular coagulation | Insufficient information /  Malignancy related | Insufficient information | 2/5 | Third-person adjudication; structured report information was insufficient for attribution. |
| 6 | 1537506612 | 2020q1  C1 | 67/M | durvalumab | Modules: B02, B03, B06, B07, B08, B10, B11; Flags: infection/sepsis, DIC/coagulopathy, other immune-related AE; Key PTs: Anaemia; Disseminated intravascular coagulation; Pneumocystis jirovecii pneumonia | Sepsis related /  Sepsis related | Sepsis related | 5/5 | Initial agreement; infection or sepsis was the dominant competing mechanism. |
| 7 | 142997099 | 2023q2  C1 | 70/M | pembrolizumab | Modules: B03, B10; Flags: infection/sepsis, DIC/coagulopathy; Key PTs: Disseminated intravascular coagulation; Malignant neoplasm progression | Multifactorial /  Multifactorial | Multifactorial | 3/5 | Initial agreement; multiple competing mechanisms precluded a single attribution. |
| 8 | 156874739 | 2021q2  C1 | 61/M | atezolizumab | Modules: B03, B06, B13; Flags: DIC/coagulopathy; Key PTs: Disseminated intravascular coagulation | Treatment-related immune/coagulation /  Treatment-related immune/coagulation | Treatment-related immune/coagulation | 3/5 | Initial agreement; treatment-related immune or coagulation toxicity was considered most plausible. |
| 9 | 194792622 | 2021q3  C2 | 64/M | nivolumab+  ipilimumab | Modules: B01, B02, B07, B14; Flags: infection/sepsis, DIC/coagulopathy; Key PTs: Disseminated intravascular coagulation; Pneumonia | Multifactorial /  Multifactorial | Multifactorial | 4/5 | Initial agreement; multiple competing mechanisms precluded a single attribution. |
| 10 | 208372012 | 2022q2  C1 | NA/F | pembrolizumab | Modules: B08; Flags: DIC/coagulopathy, marrow toxicity, other immune-related AE; Key PTs: Disseminated intravascular coagulation; Malignant neoplasm progression | Malignancy related /  Malignancy related | Malignancy related | 5/5 | Initial agreement; malignancy or progression was the dominant competing mechanism. |
| 11 | 132706081 | 2017q1  C1 | 55/F | pembrolizumab | Modules: B01, B03; Flags: infection/sepsis, DIC/coagulopathy; Key PTs: Anaemia; Disseminated intravascular coagulation; Malignant neoplasm progression | Multifactorial /  Sepsis related | Multifactorial | 4/5 | Third-person adjudication; multiple competing mechanisms precluded a single attribution. |
| 12 | 142618135 | 2018q1  C1 | 73/F | pembrolizumab | Modules: B02, B10; Flags: infection/sepsis, DIC/coagulopathy; Key PTs: Disseminated intravascular coagulation; Haemolytic anaemia; Haemolytic uraemic syndrome | Treatment-related immune/coagulation /  Treatment-related immune/coagulation | Treatment-related immune/coagulation | 4/5 | Initial agreement; treatment-related immune or coagulation toxicity was considered most plausible. |
| 13 | 177305516 | 2020q3  C2 | 68/M | pembrolizumab | Modules: B03, B07, B13, B15; Flags: DIC/coagulopathy, other immune-related AE; Key PTs: Disseminated intravascular coagulation; Immune-mediated hepatic disorder | Treatment-related immune/coagulation /  Treatment-related immune/coagulation | Treatment-related immune/coagulation | 4/5 | Initial agreement; treatment-related immune or coagulation toxicity was considered most plausible. |
| 14 | 1361458012 | 2019q1  C1 | 63/M | pembrolizumab | Modules: B01, B03, B04, B07, B10; Flags: infection/sepsis, DIC/coagulopathy, bleeding; Key PTs: Bacterial infection; Disseminated intravascular coagulation; Fungal infection | Sepsis related /  Sepsis related | Sepsis related | 5/5 | Initial agreement; infection or sepsis was the dominant competing mechanism. |
| 15 | 166577292 | 2019q3  C2 | 67/M | pembrolizumab | Modules: B03, B14; Flags: infection/sepsis, DIC/coagulopathy; Key PTs: Disseminated intravascular coagulation; Sepsis | Sepsis related /  Sepsis related | Sepsis related | 5/5 | Initial agreement; infection or sepsis was the dominant competing mechanism. |
| 16 | 151330882 | 2021q4  C1 | 76/M | pembrolizumab+  nivolumab | Modules: B03, B04, B09, B11, B15; Flags: infection/sepsis, DIC/coagulopathy, bleeding, other immune-related AE; Key PTs: Disseminated intravascular coagulation; Gastrointestinal haemorrhage; Immune-mediated lung disease | Multifactorial /  Multifactorial | Multifactorial | 4/5 | Initial agreement; multiple competing mechanisms precluded a single attribution. |
| 17 | 239500611 | 2024q2  C1 | 59/M | atezolizumab | Modules: B03, B06, B10, B11; Flags: DIC/coagulopathy; Key PTs: Anaemia; Coagulopathy | Malignancy related /  Malignancy related | Malignancy related | 4/5 | Initial agreement; malignancy or progression was the dominant competing mechanism. |
| 18 | 154351306 | 2019q1  C1 | 73/M | atezolizumab | Modules: B01, B09; Flags: DIC/coagulopathy, other immune-related AE; Key PTs: Disseminated intravascular coagulation | Treatment-related immune/coagulation /  Treatment-related immune/coagulation | Treatment-related immune/coagulation | 4/5 | Initial agreement; treatment-related immune or coagulation toxicity was considered most plausible. |
| 19 | 1258280110 | 2018q4  C2 | 64/F | nivolumab | Modules: B01, B09; Flags: DIC/coagulopathy, other immune-related AE; Key PTs: Disseminated intravascular coagulation; Malignant neoplasm progression | Multifactorial /  Multifactorial | Multifactorial | 4/5 | Initial agreement; multiple competing mechanisms precluded a single attribution. |
| 20 | 2292900915 | 2025q4  C1 | 75/M | nivolumab+  ipilimumab | Modules: B01, B03, B07, B08, B13, B14, B16; Flags: infection/sepsis, DIC/coagulopathy; Key PTs: Bacterial infection; Disseminated intravascular coagulation; Nephrogenic anaemia | Sepsis related /  Sepsis related | Sepsis related | 5/5 | Initial agreement; infection or sepsis was the dominant competing mechanism. |
| 21 | 218562244 | 2023q4  C2 | 50/M | pembrolizumab | Modules: B02, B03, B06, B07, B08, B09, B11, B13, B14; Flags: infection/sepsis, DIC/coagulopathy; Key PTs: Coagulopathy; Malignant neoplasm progression; Pneumonia | Sepsis related /  Sepsis related | Sepsis related | 5/5 | Initial agreement; infection or sepsis was the dominant competing mechanism. |
| 22 | 252540221 | 2025q2  C2 | 69/F | pembrolizumab | Modules: B03, B05, B10, B13; Flags: DIC/coagulopathy, other immune-related AE; Key PTs: Disseminated intravascular coagulation | Treatment-related immune/coagulation /  Treatment-related immune/coagulation | Treatment-related immune/coagulation | 4/5 | Initial agreement; treatment-related immune or coagulation toxicity was considered most plausible. |

*The review used public structured FAERS fields without access to complete clinical narratives, serial laboratory results, or bone-marrow findings. PrimaryID is the public FAERS report identifier.*

*Age is shown as reported; extreme values were not manually corrected. Key evidence summarizes prespecified modules, automated competing-cause flags, and up to three salient co-reported MedDRA preferred terms.*

**Reviewer-requested core analyses**

**TABLE S16 Broad four-state report counts by co-reported treatment context.**

| **Treatment context** | **Report state** | **n** | **Proportion** | **Context n** |
| --- | --- | --- | --- | --- |
| C1 | dic or mixed | 24 | 0.028 | 868 |
| C1 | immune like | 113 | 0.130 | 868 |
| C1 | marrow like | 144 | 0.166 | 868 |
| C1 | undifferentiated | 587 | 0.676 | 868 |
| C2 | dic or mixed | 24 | 0.028 | 859 |
| C2 | immune like | 40 | 0.047 | 859 |
| C2 | marrow like | 455 | 0.530 | 859 |
| C2 | undifferentiated | 340 | 0.396 | 859 |
| C3 | dic or mixed | 5 | 0.005 | 1,108 |
| C3 | immune like | 8 | 0.007 | 1,108 |
| C3 | marrow like | 602 | 0.543 | 1,108 |
| C3 | undifferentiated | 493 | 0.445 | 1,108 |

*Proportions use all four mutually exclusive report states within each observed context and are descriptive report composition, not incidence.*

**TABLE S17 Covariate-standardized fitted four-state report composition.**

| **Treatment context** | **Report state** | **Estimate** | **95% CI lower** | **95% CI upper** | **Context n** |
| --- | --- | --- | --- | --- | --- |
| C1 | dic or mixed | 0.027 | 0.018 | 0.038 | 868 |
| C1 | immune like | 0.130 | 0.110 | 0.151 | 868 |
| C1 | marrow like | 0.167 | 0.143 | 0.190 | 868 |
| C1 | undifferentiated | 0.676 | 0.646 | 0.705 | 868 |
| C2 | dic or mixed | 0.028 | 0.018 | 0.038 | 859 |
| C2 | immune like | 0.047 | 0.034 | 0.062 | 859 |
| C2 | marrow like | 0.529 | 0.499 | 0.561 | 859 |
| C2 | undifferentiated | 0.396 | 0.362 | 0.427 | 859 |
| C3 | dic or mixed | 0.005 | 0.001 | 0.009 | 1,108 |
| C3 | immune like | 0.008 | 0.003 | 0.013 | 1,108 |
| C3 | marrow like | 0.543 | 0.516 | 0.569 | 1,108 |
| C3 | undifferentiated | 0.445 | 0.418 | 0.473 | 1,108 |

*Estimates are mean fitted class probabilities among reports observed in the named context; 95% intervals used 1,000 context-stratified bootstrap samples. They are not causal counterfactual probabilities.*

**TABLE S18 C3 small-cell stress tests.**

| **Analysis** | **n** | **C3 immune n** | **C3 marrow n** | **C1 immune n** | **C1 marrow n** | **Adjusted OR** | **Adjusted 95% CI lower** | **Adjusted 95% CI upper** | **P value** | **Converged** |
| --- | --- | --- | --- | --- | --- | --- | --- | --- | --- | --- |
| Baseline C3 versus C1 | 867 | 8 | 602 | 113 | 144 | 0.014 | 0.006 | 0.029 | <0.001 | Yes |
| Lexically strict C3 immune-thrombocytopenia PT only | 864 | 5 | 602 | 113 | 144 | 0.011 | 0.004 | 0.024 | <0.001 | Yes |
| Exclude C3 report with malignant-neoplasm-progression PT | 866 | 7 | 602 | 113 | 144 | 0.012 | 0.005 | 0.026 | <0.001 | Yes |
| Exclude C3 reports with other explicit immune-related PTs | 864 | 5 | 602 | 113 | 144 | 0.008 | 0.003 | 0.020 | <0.001 | Yes |

*C3 immune-anchor PT composition: 5 of 8 reports contained “immune thrombocytopenia” and 3 of 8 contained “immune thrombocytopenic purpura”; this descriptive composition does not estimate mechanism prevalence.*

*Firth logistic models used C1 as reference. The C3 estimates are exploratory because only eight baseline C3 reports were immune-like.*

**TABLE S19 Stricter report-phenotype and competing-context sensitivity analyses.**

| **Analysis** | **n** | **Excluded n** | **C1 immune n** | **C1 marrow n** | **C2 immune n** | **C2 marrow n** | **Adjusted OR** | **95% CI lower** | **95% CI upper** | **P value** |
| --- | --- | --- | --- | --- | --- | --- | --- | --- | --- | --- |
| Baseline frozen anchors | 752 | 0 | 113 | 144 | 40 | 455 | 0.113 | 0.071 | 0.177 | <0.001 |
| Narrow immune-PT sensitivity | 752 | 0 | 113 | 144 | 40 | 455 | 0.113 | 0.071 | 0.177 | <0.001 |
| HCP-only sensitivity | 627 | 125 | 87 | 115 | 34 | 391 | 0.111 | 0.066 | 0.181 | <0.001 |
| Exclude literature reports | 631 | 121 | 87 | 127 | 25 | 392 | 0.104 | 0.060 | 0.174 | <0.001 |
| Competing-cause-negative sensitivity | 583 | 169 | 96 | 110 | 30 | 347 | 0.094 | 0.055 | 0.157 | <0.001 |
| Narrow plus competing-cause-negative | 583 | 169 | 96 | 110 | 30 | 347 | 0.094 | 0.055 | 0.157 | <0.001 |

*Estimates compare the relative composition of coding-defined report phenotypes; they do not estimate incidence or causal treatment effects.*

**TABLE S20 Availability of treatment and event dates.**

| **Cohort** | **Treatment context** | **Denominator** | **Event date n** | **ICI start n** | **Chemo start n** | **Both start n** | **Both starts on OR before event n** | **Both end n** | **Interval overlap evaluable n** | **Interval overlap n** |
| --- | --- | --- | --- | --- | --- | --- | --- | --- | --- | --- |
| all C1/C2 evidence | C1 | 858 | 514 | 570.000 | 0 | 0 | 0.000 | 0 | 0 | 0 |
| all C1/C2 evidence | C2 | 843 | 632 | 605.000 | 562 | 551 | 481.000 | 314 | 309 | 297 |
| mutually exclusive anchors | C1 | 257 | 130 | 144.000 | 0 | 0 | 0.000 | 0 | 0 | 0 |
| mutually exclusive anchors | C2 | 495 | 351 | 331.000 | 311 | 306 | 271.000 | 180 | 178 | 168 |

*Counts reflect recorded structured date fields. Missing dates were not imputed, and recorded overlap does not prove adherence or complete concomitant administration.*

**TABLE S21 Treatment-date compatibility sensitivity analyses.**

| **Analysis** | **n** | **C1 n** | **C2 n** | **C1 immune n** | **C1 marrow n** | **C2 immune n** | **C2 marrow n** | **Adjusted OR** | **95% CI lower** | **95% CI upper** | **P value** |
| --- | --- | --- | --- | --- | --- | --- | --- | --- | --- | --- | --- |
| Baseline frozen anchors | 752 | 257 | 495 | 113 | 144 | 40 | 455 | 0.113 | 0.071 | 0.177 | <0.001 |
| Both C2 treatment starts and event date available | 400 | 109 | 291 | 59 | 50 | 23 | 268 | 0.060 | 0.029 | 0.119 | <0.001 |
| Both C2 treatment starts on/before event | 380 | 109 | 271 | 59 | 50 | 22 | 249 | 0.060 | 0.028 | 0.120 | <0.001 |
| C2 starts within 30 days and on/before event | 366 | 109 | 257 | 59 | 50 | 18 | 239 | 0.048 | 0.021 | 0.101 | <0.001 |
| C2 starts within 90 days and on/before event | 373 | 109 | 264 | 59 | 50 | 19 | 245 | 0.050 | 0.023 | 0.104 | <0.001 |
| C2 documented treatment-interval overlap | 262 | 109 | 153 | 59 | 50 | 13 | 140 | 0.060 | 0.025 | 0.133 | <0.001 |

*Firth estimates compare coding-defined report composition under increasingly restrictive date-compatibility rules. Missing dates were not imputed.*

**TABLE S22 Locked report-phenotype model ablation performance.**

| **Model** | **Dataset** | **n** | **Immune n** | **Metric** | **Estimate** | **95% CI lower** | **95% CI upper** |
| --- | --- | --- | --- | --- | --- | --- | --- |
| Context free modules | FAERS development OOF | 604 | 133 | auroc | 0.737 | 0.697 | 0.781 |
| Context free modules | FAERS development OOF | 604 | 133 | pr auc | 0.476 | 0.409 | 0.552 |
| Context free modules | FAERS development OOF | 604 | 133 | brier | 0.148 | 0.138 | 0.157 |
| Context free modules | FAERS development OOF | 604 | 133 | log loss | 0.452 | 0.422 | 0.477 |
| Context free modules | FAERS development OOF | 604 | 133 | calibration intercept | -0.117 | -0.289 | 0.122 |
| Context free modules | FAERS development OOF | 604 | 133 | calibration slope | 0.865 | 0.706 | 1.082 |
| Context free modules | FAERS temporal | 148 | 20 | auroc | 0.760 | 0.667 | 0.845 |
| Context free modules | FAERS temporal | 148 | 20 | pr auc | 0.317 | 0.213 | 0.504 |
| Context free modules | FAERS temporal | 148 | 20 | brier | 0.107 | 0.096 | 0.116 |
| Context free modules | FAERS temporal | 148 | 20 | log loss | 0.340 | 0.305 | 0.374 |
| Context free modules | FAERS temporal | 148 | 20 | calibration intercept | 0.393 | -0.350 | 1.364 |
| Context free modules | FAERS temporal | 148 | 20 | calibration slope | 1.065 | 0.686 | 1.611 |
| Context free modules | JADER transport | 376 | 145 | auroc | 0.720 | 0.667 | 0.768 |
| Context free modules | JADER transport | 376 | 145 | pr auc | 0.607 | 0.542 | 0.678 |
| Context free modules | JADER transport | 376 | 145 | brier | 0.222 | 0.211 | 0.233 |
| Context free modules | JADER transport | 376 | 145 | log loss | 0.628 | 0.602 | 0.655 |
| Context free modules | JADER transport | 376 | 145 | calibration intercept | 0.993 | 0.652 | 1.400 |
| Context free modules | JADER transport | 376 | 145 | calibration slope | 1.353 | 1.015 | 1.734 |
| Modules only | FAERS development OOF | 604 | 133 | auroc | 0.709 | 0.666 | 0.748 |
| Modules only | FAERS development OOF | 604 | 133 | pr auc | 0.379 | 0.326 | 0.446 |
| Modules only | FAERS development OOF | 604 | 133 | brier | 0.152 | 0.145 | 0.160 |
| Modules only | FAERS development OOF | 604 | 133 | log loss | 0.462 | 0.440 | 0.487 |
| Modules only | FAERS development OOF | 604 | 133 | calibration intercept | -0.035 | -0.312 | 0.283 |
| Modules only | FAERS development OOF | 604 | 133 | calibration slope | 0.957 | 0.708 | 1.268 |
| Modules only | FAERS temporal | 148 | 20 | auroc | 0.778 | 0.688 | 0.861 |
| Modules only | FAERS temporal | 148 | 20 | pr auc | 0.307 | 0.216 | 0.492 |
| Modules only | FAERS temporal | 148 | 20 | brier | 0.107 | 0.095 | 0.119 |
| Modules only | FAERS temporal | 148 | 20 | log loss | 0.346 | 0.313 | 0.380 |
| Modules only | FAERS temporal | 148 | 20 | calibration intercept | -0.121 | -0.707 | 0.834 |
| Modules only | FAERS temporal | 148 | 20 | calibration slope | 1.326 | 0.823 | 2.300 |
| Modules only | JADER transport | 376 | 145 | auroc | 0.649 | 0.600 | 0.694 |
| Modules only | JADER transport | 376 | 145 | pr auc | 0.511 | 0.467 | 0.556 |
| Modules only | JADER transport | 376 | 145 | brier | 0.231 | 0.224 | 0.238 |
| Modules only | JADER transport | 376 | 145 | log loss | 0.649 | 0.630 | 0.668 |
| Modules only | JADER transport | 376 | 145 | calibration intercept | 0.988 | 0.551 | 1.524 |
| Modules only | JADER transport | 376 | 145 | calibration slope | 1.463 | 0.998 | 2.070 |
| Primary full context | FAERS development OOF | 604 | 133 | auroc | 0.834 | 0.796 | 0.871 |
| Primary full context | FAERS development OOF | 604 | 133 | pr auc | 0.592 | 0.522 | 0.674 |
| Primary full context | FAERS development OOF | 604 | 133 | brier | 0.125 | 0.113 | 0.138 |
| Primary full context | FAERS development OOF | 604 | 133 | log loss | 0.393 | 0.356 | 0.428 |
| Primary full context | FAERS development OOF | 604 | 133 | calibration intercept | 0.025 | -0.159 | 0.251 |
| Primary full context | FAERS development OOF | 604 | 133 | calibration slope | 1.026 | 0.857 | 1.238 |
| Primary full context | FAERS temporal | 148 | 20 | auroc | 0.756 | 0.643 | 0.861 |
| Primary full context | FAERS temporal | 148 | 20 | pr auc | 0.382 | 0.253 | 0.615 |
| Primary full context | FAERS temporal | 148 | 20 | brier | 0.103 | 0.084 | 0.123 |
| Primary full context | FAERS temporal | 148 | 20 | log loss | 0.343 | 0.289 | 0.409 |
| Primary full context | FAERS temporal | 148 | 20 | calibration intercept | -0.454 | -1.008 | 0.373 |
| Primary full context | FAERS temporal | 148 | 20 | calibration slope | 0.854 | 0.469 | 1.512 |
| Primary full context | JADER transport | 376 | 145 | auroc | 0.880 | 0.841 | 0.914 |
| Primary full context | JADER transport | 376 | 145 | pr auc | 0.823 | 0.766 | 0.876 |
| Primary full context | JADER transport | 376 | 145 | brier | 0.161 | 0.144 | 0.179 |
| Primary full context | JADER transport | 376 | 145 | log loss | 0.489 | 0.439 | 0.539 |
| Primary full context | JADER transport | 376 | 145 | calibration intercept | 1.313 | 0.982 | 1.747 |
| Primary full context | JADER transport | 376 | 145 | calibration slope | 1.328 | 1.091 | 1.614 |
| Secondary full context modules | FAERS development OOF | 604 | 133 | auroc | 0.862 | 0.827 | 0.892 |
| Secondary full context modules | FAERS development OOF | 604 | 133 | pr auc | 0.642 | 0.572 | 0.723 |
| Secondary full context modules | FAERS development OOF | 604 | 133 | brier | 0.117 | 0.104 | 0.130 |
| Secondary full context modules | FAERS development OOF | 604 | 133 | log loss | 0.365 | 0.330 | 0.399 |
| Secondary full context modules | FAERS development OOF | 604 | 133 | calibration intercept | 0.019 | -0.157 | 0.228 |
| Secondary full context modules | FAERS development OOF | 604 | 133 | calibration slope | 1.007 | 0.845 | 1.208 |
| Secondary full context modules | FAERS temporal | 148 | 20 | auroc | 0.830 | 0.730 | 0.913 |
| Secondary full context modules | FAERS temporal | 148 | 20 | pr auc | 0.454 | 0.314 | 0.671 |
| Secondary full context modules | FAERS temporal | 148 | 20 | brier | 0.097 | 0.077 | 0.119 |
| Secondary full context modules | FAERS temporal | 148 | 20 | log loss | 0.317 | 0.260 | 0.388 |
| Secondary full context modules | FAERS temporal | 148 | 20 | calibration intercept | -0.457 | -0.899 | 0.320 |
| Secondary full context modules | FAERS temporal | 148 | 20 | calibration slope | 0.900 | 0.551 | 1.580 |
| Secondary full context modules | JADER transport | 376 | 145 | auroc | 0.894 | 0.857 | 0.925 |
| Secondary full context modules | JADER transport | 376 | 145 | pr auc | 0.827 | 0.767 | 0.882 |
| Secondary full context modules | JADER transport | 376 | 145 | brier | 0.147 | 0.131 | 0.165 |
| Secondary full context modules | JADER transport | 376 | 145 | log loss | 0.449 | 0.406 | 0.494 |
| Secondary full context modules | JADER transport | 376 | 145 | calibration intercept | 1.105 | 0.799 | 1.520 |
| Secondary full context modules | JADER transport | 376 | 145 | calibration slope | 1.385 | 1.146 | 1.688 |

*Development estimates use out-of-fold predictions. Temporal and JADER sets were evaluated without refitting, feature selection, threshold reselection, or recalibration. Metrics describe coding-defined report-phenotype discrimination and calibration, not clinical diagnostic performance.*

**TABLE S23 Score distribution after treatment-context and drug-class ablation.**

| **Model** | **Treatment context** | **n** | **Mean probability** | **Median probability** | **Low lt 0 30 n** | **Intermediate 0 30 to 0 70 n** | **High gt 0 70 n** |
| --- | --- | --- | --- | --- | --- | --- | --- |
| Primary full context | C1 | 587 | 0.469 | 0.481 | 104 | 444 | 39 |
| Primary full context | C2 | 340 | 0.104 | 0.093 | 334 | 6 | 0 |
| Secondary full context modules | C1 | 587 | 0.462 | 0.470 | 109 | 435 | 43 |
| Secondary full context modules | C2 | 340 | 0.119 | 0.096 | 326 | 14 | 0 |
| Context free modules | C1 | 587 | 0.277 | 0.259 | 352 | 231 | 4 |
| Context free modules | C2 | 340 | 0.216 | 0.219 | 265 | 75 | 0 |
| Modules only | C1 | 587 | 0.250 | 0.312 | 280 | 307 | 0 |
| Modules only | C2 | 340 | 0.256 | 0.312 | 148 | 192 | 0 |

*Probabilities are report-phenotype scores. Thresholds define descriptive score bands and have no diagnostic meaning.*

**TABLE S24 Report-level onset-proxy window sensitivity analyses.**

| **Window** | **Window n** | **Immune n** | **Immune median** | **Immune q1** | **Immune q3** | **Marrow n** | **Marrow median** | **Marrow q1** | **Marrow q3** | **Median difference immune minus marrow** | **Median difference CI lower** | **Median difference CI upper** | **Cliffs delta** | **Cliffs delta CI lower** | **Cliffs delta CI upper** | **P value two sided** |
| --- | --- | --- | --- | --- | --- | --- | --- | --- | --- | --- | --- | --- | --- | --- | --- | --- |
| full valid 0 to 780 | 403 | 84 | 27.500 | 11.000 | 81.750 | 319 | 10.000 | 6.000 | 21.500 | 17.500 | 9.000 | 36.000 | 0.390 | 0.254 | 0.525 | 0.00 |
| truncated 0 to 180 | 385 | 74 | 21.000 | 10.000 | 59.250 | 311 | 10.000 | 5.500 | 20.000 | 11.000 | 5.000 | 19.000 | 0.346 | 0.198 | 0.485 | 0.00 |
| truncated 0 to 365 | 397 | 82 | 26.000 | 11.000 | 71.500 | 315 | 10.000 | 6.000 | 21.000 | 16.000 | 8.000 | 33.000 | 0.393 | 0.249 | 0.526 | 0.00 |
| exclude day zero full range | 387 | 78 | 28.000 | 14.000 | 90.750 | 309 | 10.000 | 6.000 | 24.000 | 18.000 | 11.000 | 41.500 | 0.478 | 0.355 | 0.594 | 0 |

*Intervals are report-level onset proxies derived from incomplete spontaneous-report dates, not clinical latency estimates. Median-difference and Cliff's delta intervals used 5,000 bootstrap samples.*

**Final random stratified blinded review and adjudication**

**TABLE S25 Agreement between the two independent reviewers before adjudication.**

| **Estimand** | **Metric** | **Estimate** | **95% CI lower** | **95% CI upper** | **Bootstrap replicates** |
| --- | --- | --- | --- | --- | --- |
| sample unweighted | exact agreement | 0.925 | 0.875 | 0.967 | 5,000 |
| sample unweighted | cohen kappa | 0.897 | 0.831 | 0.954 | 5,000 |
| sample unweighted | linear weighted kappa sensitivity | 0.940 | 0.901 | 0.974 | 5,000 |
| sample unweighted | gwet ac1 | 0.901 | 0.835 | 0.956 | 5,000 |
| design weighted to 927 frame | exact agreement | 0.932 | 0.885 | 0.971 | 5,000 |
| design weighted to 927 frame | cohen kappa | 0.906 | 0.841 | 0.960 | 5,000 |
| design weighted to 927 frame | linear weighted kappa sensitivity | 0.944 | 0.904 | 0.977 | 5,000 |
| design weighted to 927 frame | gwet ac1 | 0.910 | 0.849 | 0.962 | 5,000 |

*Exact agreement, Cohen's κ, linear-weighted κ, and Gwet's AC1 were calculated before adjudication. Intervals use 5,000 bootstrap samples within the six locked sampling strata.*

**TABLE S26 Four-category cross-classification before adjudication.**

| **Reviewer a** | **Reviewer b** | **n** |
| --- | --- | --- |
| Immune probable | Immune probable | 21 |
| Immune probable | Immune possible | 4 |
| Immune probable | Indeterminate | 0 |
| Immune probable | Non-immune probable | 0 |
| Immune possible | Immune probable | 0 |
| Immune possible | Immune possible | 32 |
| Immune possible | Indeterminate | 5 |
| Immune possible | Non-immune probable | 0 |
| Indeterminate | Immune probable | 0 |
| Indeterminate | Immune possible | 0 |
| Indeterminate | Indeterminate | 18 |
| Indeterminate | Non-immune probable | 0 |
| Non-immune probable | Immune probable | 0 |
| Non-immune probable | Immune possible | 0 |
| Non-immune probable | Indeterminate | 0 |
| Non-immune probable | Non-immune probable | 40 |

*Rows are Reviewer A ratings and columns are Reviewer B ratings. The table includes all 120 randomly stratified reports before third-person adjudication.*

**TABLE S27 Class-specific agreement before adjudication.**

| **Class** | **Reviewer a n** | **Reviewer b n** | **Both n** | **Class specific positive agreement** |
| --- | --- | --- | --- | --- |
| Immune probable | 25 | 21 | 21 | 0.91 |
| Immune possible | 37 | 36 | 32 | 0.88 |
| Indeterminate | 18 | 23 | 18 | 0.88 |
| Non-immune probable | 40 | 40 | 40 | 1 |

*Class-specific positive agreement is descriptive and was calculated before adjudication.*

**TABLE S28 Reports with discordant independent ratings requiring adjudication.**

| **Review id** | **PrimaryID** | **Source period** | **Final judgment A** | **Confidence (1-5) A** | **Primary reason code A** | **Final judgment B** | **Confidence (1-5) B** | **Primary reason code B** |
| --- | --- | --- | --- | --- | --- | --- | --- | --- |
| CR-40E4FC3016 | 190203822 | 2021q1 | Immune possible | 4 | Other immune-related event supportive | Indeterminate | 2 | Insufficient report information |
| CR-4A3E3A86F7 | 264213941 | 2026q1 | Immune probable | 5 | Isolated thrombocytopenia pattern | Immune possible | 2 | Isolated thrombocytopenia pattern |
| CR-4ABCB657E2 | 193816581 | 2021q2 | Immune probable | 4 | Other immune-related event supportive | Immune possible | 3 | Other immune-related event supportive |
| CR-4BEFDE4F8D | 246058688 | 2025q4 | Immune probable | 4 | Temporal pattern supportive | Immune possible | 2 | Temporal pattern supportive |
| CR-732BCFCD12 | 232188092 | 2023q4 | Immune possible | 3 | Other immune-related event supportive | Indeterminate | 2 | Insufficient report information |
| CR-7784BE9662 | 150898194 | 2019q1 | Immune probable | 4 | Temporal pattern supportive | Immune possible | 2 | Temporal pattern supportive |
| CR-C7058D603B | 143662891 | 2018q1 | Immune possible | 2 | Isolated thrombocytopenia pattern | Indeterminate | 2 | Insufficient report information |
| CR-DCE4BF7538 | 245525802 | 2025q3 | Immune possible | 2 | Isolated thrombocytopenia pattern | Indeterminate | 2 | Insufficient report information |
| CR-E18268A524 | 218855963 | 2023q1 | Immune possible | 3 | Other immune-related event supportive | Indeterminate | 2 | Insufficient report information |

*All nine disagreements were between adjacent review categories. Treatment-context labels, model probabilities, ranks, and sampling-stratum labels were not shown in the reviewer workbooks.*

**TABLE S29 Final consensus distribution after third-person adjudication.**

| **Final consensus** | **Sample n** | **Design weighted count** | **Sample proportion** | **Design weighted proportion** | **Design weighted CI lower** | **Design weighted CI upper** |
| --- | --- | --- | --- | --- | --- | --- |
| Immune probable | 23 | 216.65 | 0.192 | 0.234 | 0.158 | 0.308 |
| Immune possible | 37 | 345.55 | 0.308 | 0.373 | 0.293 | 0.461 |
| Indeterminate | 20 | 125.70 | 0.167 | 0.136 | 0.084 | 0.195 |
| Non-immune probable | 40 | 239.10 | 0.333 | 0.258 | 0.205 | 0.316 |

*Design weights are the inverse sampling fractions for the six locked C1/C2 by within-context score-tertile strata. Intervals use 2,000 within-stratum bootstrap samples. Categories are structured-report judgments, not confirmed diagnoses.*

**TABLE S30 Final consensus categories within each locked sampling stratum.**

| **Treatment context** | **Score tertile number** | **Score tertile** | **Stratum population n** | **Sample n** | **Final consensus** | **Category n** | **Category proportion** |
| --- | --- | --- | --- | --- | --- | --- | --- |
| C1 | 1.000 | lower within context | 196 | 20 | Immune probable | 5 | 0.250 |
| C1 | 1.000 | lower within context | 196 | 20 | Immune possible | 12 | 0.600 |
| C1 | 1.000 | lower within context | 196 | 20 | Indeterminate | 1 | 0.050 |
| C1 | 1.000 | lower within context | 196 | 20 | Non-immune probable | 2 | 0.100 |
| C1 | 2.000 | middle within context | 196 | 20 | Immune probable | 6 | 0.300 |
| C1 | 2.000 | middle within context | 196 | 20 | Immune possible | 11 | 0.550 |
| C1 | 2.000 | middle within context | 196 | 20 | Indeterminate | 2 | 0.100 |
| C1 | 2.000 | middle within context | 196 | 20 | Non-immune probable | 1 | 0.050 |
| C1 | 3.000 | upper within context | 195 | 20 | Immune probable | 10 | 0.500 |
| C1 | 3.000 | upper within context | 195 | 20 | Immune possible | 10 | 0.500 |
| C1 | 3.000 | upper within context | 195 | 20 | Indeterminate | 0 | 0.000 |
| C1 | 3.000 | upper within context | 195 | 20 | Non-immune probable | 0 | 0.000 |
| C2 | 1.000 | lower within context | 114 | 20 | Immune probable | 1 | 0.050 |
| C2 | 1.000 | lower within context | 114 | 20 | Immune possible | 1 | 0.050 |
| C2 | 1.000 | lower within context | 114 | 20 | Indeterminate | 5 | 0.250 |
| C2 | 1.000 | lower within context | 114 | 20 | Non-immune probable | 13 | 0.650 |
| C2 | 2.000 | middle within context | 113 | 20 | Immune probable | 1 | 0.050 |
| C2 | 2.000 | middle within context | 113 | 20 | Immune possible | 2 | 0.100 |
| C2 | 2.000 | middle within context | 113 | 20 | Indeterminate | 6 | 0.300 |
| C2 | 2.000 | middle within context | 113 | 20 | Non-immune probable | 11 | 0.550 |
| C2 | 3.000 | upper within context | 113 | 20 | Immune probable | 0 | 0.000 |
| C2 | 3.000 | upper within context | 113 | 20 | Immune possible | 1 | 0.050 |
| C2 | 3.000 | upper within context | 113 | 20 | Indeterminate | 6 | 0.300 |
| C2 | 3.000 | upper within context | 113 | 20 | Non-immune probable | 13 | 0.650 |

*Twenty reports were randomly sampled from each of six strata using seed 20260826. Stratum proportions describe the reviewed sample and should not be read as unweighted population prevalence.*

**TABLE S31 Report-phenotype score distribution by final consensus category.**

| **Final consensus** | **Sample n** | **Design weighted mean score** | **Design weighted q1** | **Design weighted median** | **Design weighted q3** | **Score min** | **Score max** |
| --- | --- | --- | --- | --- | --- | --- | --- |
| Immune probable | 23 | 0.475 | 0.382 | 0.473 | 0.650 | 0.062 | 0.706 |
| Immune possible | 37 | 0.417 | 0.279 | 0.431 | 0.594 | 0.008 | 0.807 |
| Indeterminate | 20 | 0.199 | 0.063 | 0.138 | 0.282 | 0.019 | 0.550 |
| Non-immune probable | 40 | 0.128 | 0.055 | 0.105 | 0.188 | 0.004 | 0.485 |

*Scores are locked report-phenotype outputs. Weighted summaries use inverse sampling fractions and do not represent calibrated diagnostic probabilities.*

**TABLE S32 Exploratory agreement between the locked score and final review categories.**

| **Definition** | **n** | **Positive n** | **Negative n** | **Metric** | **Estimate** | **95% CI lower** | **95% CI upper** | **Successful bootstrap n** |
| --- | --- | --- | --- | --- | --- | --- | --- | --- |
| primary determinate | 100 | 60 | 40 | weighted positive proportion | 0.702 | 0.647 | 0.756 | 2,000 |
| primary determinate | 100 | 60 | 40 | auroc | 0.892 | 0.827 | 0.948 | 2,000 |
| primary determinate | 100 | 60 | 40 | pr auc | 0.956 | 0.922 | 0.982 | 2,000 |
| primary determinate | 100 | 60 | 40 | brier | 0.256 | 0.220 | 0.293 | 2,000 |
| primary determinate | 100 | 60 | 40 | calibration in large intercept | 2.054 | 1.707 | 2.432 | 2,000 |
| primary determinate | 100 | 60 | 40 | recalibration intercept | 2.536 | 1.938 | 3.666 | 1,876 |
| primary determinate | 100 | 60 | 40 | calibration slope | 1.345 | 0.997 | 2.181 | 1,876 |
| strict probable | 63 | 23 | 40 | weighted positive proportion | 0.475 | 0.366 | 0.569 | 2,000 |
| strict probable | 63 | 23 | 40 | auroc | 0.920 | 0.837 | 0.985 | 2,000 |
| strict probable | 63 | 23 | 40 | pr auc | 0.932 | 0.855 | 0.988 | 2,000 |
| strict probable | 63 | 23 | 40 | brier | 0.162 | 0.112 | 0.210 | 2,000 |
| strict probable | 63 | 23 | 40 | calibration in large intercept | 1.092 | 0.638 | 1.516 | 2,000 |
| strict probable | 63 | 23 | 40 | recalibration intercept | 2.030 | 1.175 | 4.192 | 1,989 |
| strict probable | 63 | 23 | 40 | calibration slope | 1.856 | 1.191 | 4.107 | 1,989 |
| conservative indeterminate negative | 120 | 60 | 60 | weighted positive proportion | 0.606 | 0.550 | 0.659 | 2,000 |
| conservative indeterminate negative | 120 | 60 | 60 | auroc | 0.869 | 0.806 | 0.929 | 2,000 |
| conservative indeterminate negative | 120 | 60 | 60 | pr auc | 0.918 | 0.873 | 0.962 | 2,000 |
| conservative indeterminate negative | 120 | 60 | 60 | brier | 0.231 | 0.198 | 0.263 | 2,000 |
| conservative indeterminate negative | 120 | 60 | 60 | calibration in large intercept | 1.593 | 1.258 | 1.916 | 2,000 |
| conservative indeterminate negative | 120 | 60 | 60 | recalibration intercept | 1.862 | 1.331 | 2.693 | 1,995 |
| conservative indeterminate negative | 120 | 60 | 60 | calibration slope | 1.223 | 0.843 | 1.889 | 1,995 |

*Primary determinate: immune probable or possible versus non-immune probable. Strict probable: immune probable versus non-immune probable. Conservative: indeterminate treated as review-negative. Estimates use design weights and 2,000 within-stratum bootstrap samples; they are not clinical diagnostic performance.*

**TABLE S33 Third-person adjudication of the nine disagreements.**

| **Review ID** | **FAERS PrimaryID** | **Reviewer A judgment** | **Reviewer B judgment** | **Adjudicated final judgment** | **Confidence (1-5)** | **Adjudication rationale** |
| --- | --- | --- | --- | --- | --- | --- |
| CR-4A3E3A86F7 | 264213941 | Immune probable | Immune possible | Immune possible | 3 | Atezolizumab is the only reported anticancer suspect and the reaction pattern is isolated thrombocytopenia with petechiae/epistaxis, which is compatible with immune thrombocytopenia. However, treatment-to-event timing and hematologic work-up are not available, so the evidence does not support a 'probable' classification. |
| CR-4ABCB657E2 | 193816581 | Immune probable | Immune possible | Immune probable | 4 | The platelet decrease followed a recent atezolizumab exposure by about 7 days, whereas the recorded carboplatin/paclitaxel exposure was remote (~566 days). Co-reported colitis adds independent immune-toxicity support. Prior chemotherapy remains a competing cause, but the visible report evidence overall favors an immune phenotype. |
| CR-4BEFDE4F8D | 246058688 | Immune probable | Immune possible | Immune probable | 4 | The event is temporally closer to ipilimumab/nivolumab exposure (~2 days) than to the recorded cisplatin/pemetrexed course (~88 days), with additional inflammatory features including rash, pyrexia and cytokine-release syndrome. Chemotherapy is a plausible alternative, but is less compelling temporally in this report. |
| CR-732BCFCD12 | 232188092 | Immune possible | Indeterminate | Indeterminate | 3 | Durvalumab/tremelimumab exposure and adrenal insufficiency support an immune mechanism, but cisplatin/pemetrexed and concurrent COVID-19 provide substantial non-immune alternatives. With no usable onset timing or platelet/marrow/coagulation course, neither mechanism clearly predominates. |
| CR-40E4FC3016 | 190203822 | Immune possible | Indeterminate | Immune possible | 3 | Recent atezolizumab exposure and co-reported hypothyroidism keep an immune mechanism plausible, with the event appearing closer to ICI exposure than to the earlier platinum/etoposide course. However, the broader cytotoxic-treatment history and anemia/marrow-suppression context prevent a confident immune-predominant classification. |
| CR-E18268A524 | 218855963 | Immune possible | Indeterminate | Indeterminate | 3 | Pembrolizumab and carboplatin/paclitaxel were recorded together, and the available dates do not provide a reliable treatment-to-event sequence. The immune-mediated hepatic event supports immune susceptibility, but it does not establish the mechanism of thrombocytopenia in the presence of strongly myelosuppressive chemotherapy. |
| CR-DCE4BF7538 | 245525802 | Immune possible | Indeterminate | Immune possible | 2 | Durvalumab is the only reported anticancer drug and thrombocytopenia is otherwise isolated, with no cytotoxic chemotherapy recorded. This supports an immune mechanism, but the absence of usable chronology, platelet trend, treatment response or marrow/coagulation data limits the case to 'possible'. |
| CR-7784BE9662 | 150898194 | Immune probable | Immune possible | Immune possible | 3 | Platelet decrease occurred about 16 days after atezolizumab and substantially later after the recorded cisplatin/docetaxel exposure (~106 days), which favors an ICI-related mechanism. Nevertheless, the report contains a background of granulocytopenia/leukopenia and supportive hematopoietic treatment, so chemotherapy-related marrow toxicity remains credible. |
| CR-C7058D603B | 143662891 | Immune possible | Indeterminate | Immune possible | 2 | Pembrolizumab is the only reported anticancer suspect and the reported reaction is isolated platelet-count decrease without a concurrent cytotoxic regimen. In the absence of an event date, platelet course or diagnostic work-up, immune thrombocytopenia is plausible but cannot be rated 'probable'. |

*Final adjudication outcomes are shown; reviewer identity and adjudication time are omitted as specified. Results did not retrain or recalibrate the model.*
